# Ozone tolerant maize hybrids maintain Rubisco content and activity during long‐term exposure in the field

**DOI:** 10.1111/pce.13876

**Published:** 2020-10-22

**Authors:** Nicole E. Choquette, Elizabeth A. Ainsworth, William Bezodis, Amanda P. Cavanagh

**Affiliations:** ^1^ Carl R. Woese Institute for Genomic Biology University of Illinois at Urbana‐Champaign Champaign Illinois USA; ^2^ Department of Plant Biology University of Illinois at Urbana‐Champaign Champaign Illinois USA; ^3^ Global Change and Photosynthesis Research Unit USDA ARS Urbana Illinois USA; ^4^ Department of Plant Sciences University of Oxford Oxford UK; ^5^ School of Life Sciences University of Essex Colchester UK

**Keywords:** antioxidant content, climate change, nitrogen, ozone, photosynthesis, ribulose‐1,5‐bisphosphate carboxylase‐oxygenase, *Zea mays*

## Abstract

Ozone pollution is a damaging air pollutant that reduces maize yields equivalently to nutrient deficiency, heat, and aridity stress. Therefore, understanding the physiological and biochemical responses of maize to ozone pollution and identifying traits predictive of ozone tolerance is important. In this study, we examined the physiological, biochemical and yield responses of six maize hybrids to elevated ozone in the field using Free Air Ozone Enrichment. Elevated ozone stress reduced photosynthetic capacity, in vivo and in vitro, decreasing Rubisco content, but not activation state. Contrary to our hypotheses, variation in maize hybrid responses to ozone was not associated with stomatal limitation or antioxidant pools in maize. Rather, tolerance to ozone stress in the hybrid B73 × Mo17 was correlated with maintenance of leaf N content. Sensitive lines showed greater ozone‐induced senescence and loss of photosynthetic capacity compared to the tolerant line.

## Abstract 2

Ozone pollution significantly reduces maize yield, and understanding the responses underpinning ozone tolerance is critical to developing varieties to mitigate losses. In field‐grown maize, tolerance to elevated ozone is associated with increased leaf N and Rubisco content, maintaining carboxylation capacity.

## 1 INTRODUCTION

Ozone (O_3_) pollution formed in the troposphere compromises yields of many crop species and is estimated to reduce maize yields by as much as 10–15% (Avnery, Mauzerall, Liu, & Horowitz, 2011; McGrath et al., 2015; Mills et al., 2018c; Van Dingenen et al., 2009). Ozone is a short‐lived pollutant and concentrations are dynamic and variable across the globe. The highest ozone concentrations are measured in the mid‐latitudes of the northern hemisphere, with lower concentrations measured in Australia, New Zealand and southern parts of South America (Mills et al., 2018). High concentrations of ozone are measured in important crop‐growing regions in the United States, the Mediterranean region, India and China (Mills, Pleijel, et al., 2018). Physiological responses of crops to O_3_ pollution include visible injury, reduced carbon assimilation and premature leaf senescence (Ainsworth, 2017). These responses are likely interlinked and scale from the cell to the leaf to the crop canopy, negatively impacting economic yields (Emberson et al., 2018). Many studies have investigated the mechanisms of O_3_ stress on a variety of C_3_ crops, which have been widely reviewed (Ainsworth, Yendrek, Sitch, Collins, & Emberson, 2012; Ashmore, 2005; Feng, Kobayashi, & Ainsworth, 2008; Fiscus, Brooker, & Burkey, 2005; Morgan, Ainsworth, & Long, 2003). However, fewer studies investigated the physiological impacts of O_3_ stress on C_4_ plants, including maize, the most widely produced grain crop in the world (FAO, 2018), in part because early studies showed that C_4_ crops were more O_3_ tolerant than C_3_ crops (Heagle et al., 1988; Miller, 1988). Despite this, more recent modeling studies have predicted significant impacts of O_3_ on C_4_ crops (Avnery et al., 2011; McGrath et al., 2015; Mills et al., 2018b; Mills, Sharps, et al., 2018c; Van Dingenen et al., 2009), and experimental studies have shown that C_4_ plants are also sensitive to O_3_ pollution (Grantz & Vu, 2009; Grantz, Vu, Tew, & Veremis, 2012; Leisner & Ainsworth, 2012; Leitao, Bethenod, & Biolley, 2007; Leitao, Maoret, & Biolley, 2007; Li, Courbet, Ourry, & Ainsworth, 2019; Yendrek et al., 2017; Yendrek et al., 2017).

Ozone damage primarily occurs once O_3_ diffuses into the leaf through the stomata and into the apoplast. There, O_3_ reacts with the aqueous layers to form other reactive oxygen species (ROS), such as hydrogen peroxide, superoxide and hydroxyl radical (Heath, 2008). Apoplastic antioxidants, including ascorbate, glutathione and phenolic compounds, quench ROS, but if the ROS exceed the antioxidant‐quenching capacity of the apoplast, reactions in the plasma membrane can occur, along with signalling cascades that cause metabolic changes within the cell (Luwe, Takahama, & Heber, 1993). In many species, underlying differences in the content of antioxidant compounds correlated with O_3_ sensitivity (Betzelberger et al., 2010; Wellburn & Wellburn, 1996; Li, Calatayud, Gao, Uddling, & Feng, 2016), and in tropical maize, phenolic compounds, flavonoids and anthocyanin pigments increased with O_3_ stress (Singh, Agrawal, Shahi, & Agrawal, 2014). However, it has proven difficult to generalize antioxidant responses to elevated O_3_ across species and even genotypes within a species in part because the requirement for detoxification depends upon the amount of O_3_ entering leaves, which can change with stomatal responses to elevated O_3_ (Wellburn & Wellburn, 1996). Antioxidant compounds are also constantly changing and present in different cellular compartments at different concentrations, which complicate generalizations. Yet, development of accurate flux‐based models of O_3_ effects on crops requires fundamental knowledge of both stomatal behavior and detoxification capacity (Emberson et al., 2018).

Accelerated senescence and reduced photosynthetic carbon assimilation are two major determinants of crop yield loss to O_3_ pollution. Field experiments with maize, soybean and wheat have provided evidence that loss of photosynthetic capacity is a repercussion of accelerated leaf senescence in elevated O_3_ (Morgan, Bernacchi, Ort, & Long, 2004; Feng, Pang, Kobayashi, Zhu, & Ort, 2011; Yendrek, Erice, et al., 2017). Degradation of Rubisco protein and reduced Rubisco activity measured in vitro and in vivo in response to O_3_ stress has been observed in C_3_ crops (Enyedi, Eckardt, & Pell, 1992; Goumenaki, Taybi, Borland, & Barnes, 2010; Junqua et al., 2000). In C_4_ crops, Rubisco is located in the bundle sheath cells, and, therefore, may be more isolated from O_3_‐induced ROS. However, previous work indicated that bundle sheath proteins are more susceptible to oxidative damage than mesophyll cell proteins (Kingston‐Smith & Foyer, 2000), and Rubisco activity and transcript levels were significantly reduced in sugarcane (Grantz et al., 2012), switchgrass (Li et al., 2019) and juvenile maize (Leitao et al., 2007, b) exposed to elevated O_3_. Our previous research revealed significant genetic variation in the photosynthetic response of maize hybrids and indicated that the mechanisms of response to O_3_ may also vary among diverse hybrids (Choquette et al., 2019).

This study further investigates physiological and biochemical responses of six maize hybrids containing parents Hp301 and NC338, which previously exhibited greater photosynthetic sensitivity to elevated O_3_ (Choquette et al., 2019). These hybrids were grown in elevated O_3_ using Free Air Concentration Enrichment (FACE), which enables crops to be grown under field conditions, but with an altered atmospheric composition. Specifically, we test for genetic variation in O_3_ response by examining the effects of elevated O_3_ on the photosynthetic capacity in vivo and in vitro, stomatal limitation to photosynthesis, antioxidant pools and nitrogen (N) content. Based on previous experiments of midday gas exchange (Choquette et al., 2019), we predict that variation in sensitivity to elevated O_3_ will be correlated to differential stomatal responses to O_3_ stress, as well as to differences in antioxidant stores.

## 2 MATERIALS AND METHODS

### 2.1 Field site and ozone fumigation

**FIGURE 1:**
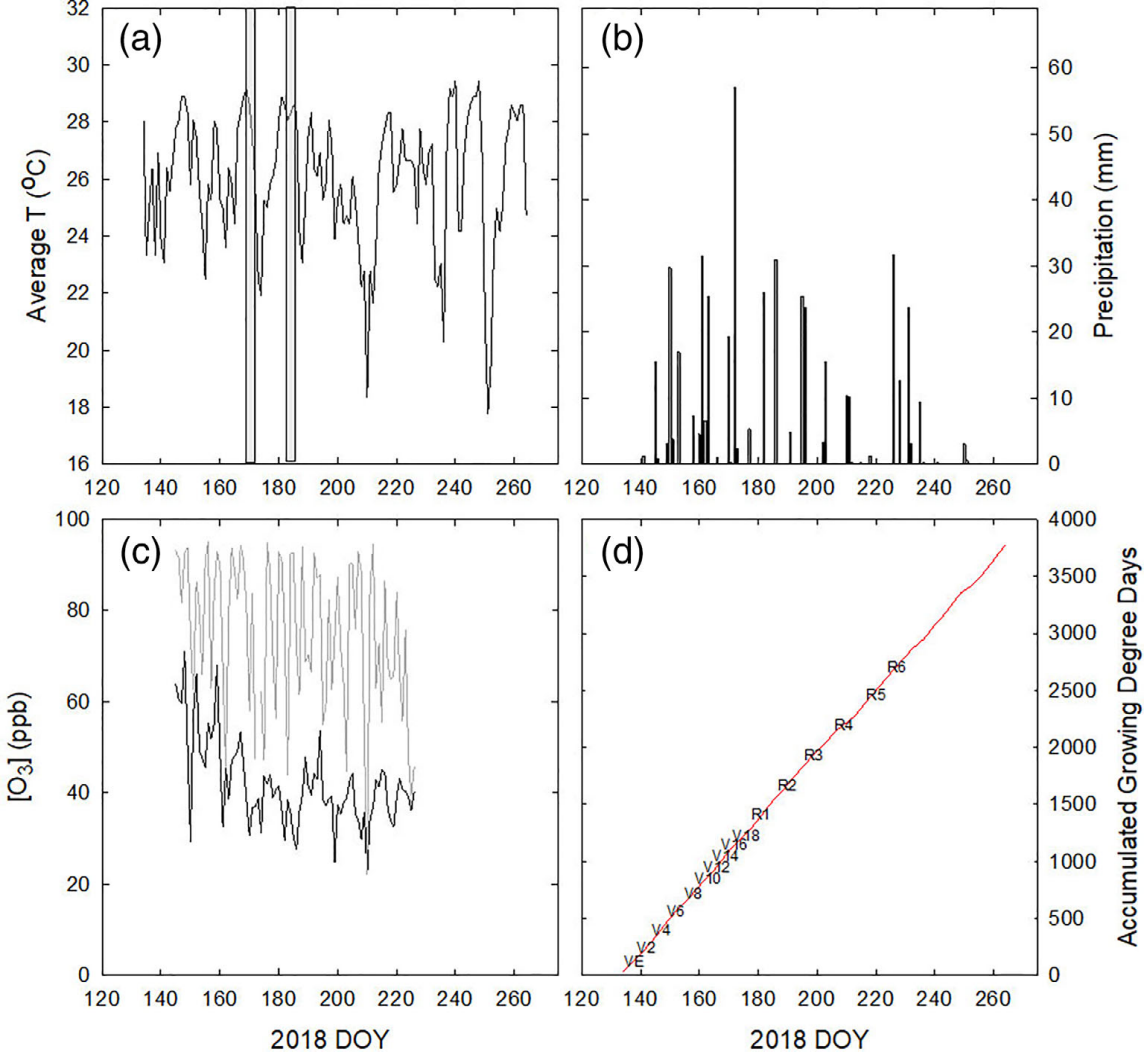
Average temperature (a), total precipitation (b), average O_3_ in ambient (black line) and elevated O_3_ plots (grey line) (c), and calculated growing degree days with estimated developmental stages from emergence (VE) through maturity (R6) (d) in 2018. Vegetative stages estimate the number of leaves with a collar. Reproductive stages are defined as R1 (silking), R2 (blistering), R3 (milk), R4 (dough), R5 (dent) and R6 (physiological maturity). Vertical grey bars in panel (a) indicate the dates of gas exchange experiments and sampling for biochemical analysis

Maize F1 hybrids B73 × Hp301, B73 × Mo17, B73 × NC338, Mo17 × Hp301, Mo17 × NC338, and NC338 × Hp301 were planted on May 13, 2018 at the FACE facility near Champaign, IL (40°02′ N, 88°14′ W, https://soyface.illinois.edu/). Within experimental and control plots, each genotype was planted in two 3.5 m rows spaced 0.76 m apart. Plant density was ~8 plants m^−1^. The six maize genotypes occupied one half of each 20 m diameter octagonal plot and were exposed to either ambient or elevated O_3_. The layout of the experiment was a randomized complete block design with *n* = 4. The O_3_ target set‐point was 100 nl L^−1^ and was applied from 10:00 to 18:00 throughout the growing season, as described in Yendrek, Tomaz, et al. (2017). In 2018, the 1 min. average O_3_ concentration within the elevated plots was within 20% of the target concentration for 81.6% of the time. When it was raining, leaves were wet, or the wind speed was lower than 0.5 m s^−1^, the O_3_ treatment was not applied. Average temperature and precipitation from the growing season were recorded in an on‐site weather station, and average developmental stages were estimated from growing degree days (Figure 1).

### 2.2 Gas exchange measurements

Gas exchange was measured from June 18–21, 2018 to July 2–5, 2018 on the eighth leaf, which was the youngest fully expanded leaf in the June measurements. The eighth leaf was tagged for tissue sampling and gas exchange measurements for both time points. Measuring the same leaf number in two time points provided information about the cumulative effects of chronic O_3_ exposure on photosynthetic and biochemical mechanisms over time. Leaves from one block of the experiment (i.e., one ambient and one elevated O_3_ plot) were excised before dawn for measurement in a field laboratory. Leaves were recut under water and placed in 50 ml tubes filled with water. Before starting gas exchange measurements, leaves were placed under grow lights (YG 600 W Grow Light, YGROW) with light spectrum of 380–740 nm for ~20 min to acclimate to high light before starting gas exchange measurements. Leaves were then placed in the leaf cuvette of a portable gas exchange system (LI‐6800, LICOR, Lincoln, NE) to measure the response of net carbon assimilation (*A*) to intercellular CO_2_ (c_i_). Once steady‐state values of *A* and g_s_ (at 400 μmol mol^−1^ CO_2_, 1800 μmol m^−2^ s^−1^ PPFD, 30.0°C leaf temperature and 1.5 kPa vapour pressure deficit) were reached, measurements were taken at 400, 300, 200, 100, 10, 400, 400, 600, 800, 1,000, 1,200, and 400 μmol mol^−1^ CO_2_. After the *A*/c_i_ response curves finished, leaves were left to reach steady state at 400 μmol mol^−1^. Once *A*/c_i_ curves reached steady state at 400 μmol mol^−1^, three leaf disks of 1.46 cm^2^ from the portion of the leaf that was enclosed in the cuvette were cut, snap‐frozen in liquid N for later quantification of Rubisco content and activation status.

From the *A*/c_i_ response curves, the maximum apparent rate of phospho*enol*pyruvate (PEP) carboxylase activity (*V*_*pmax*_) and CO2‐saturated photosynthetic rate (*V*_*max*_) were estimated. Two leaves per genotype per plot per time point were measured for a total of 192 measurements. The initial slope of the *A*/c_i_ curve was used to calculate *V*_*pmax*_ according to von Caemmerer (2000). A four‐parameter nonrectangular hyperbolic function was used to estimate *V*_*max*_ as the horizontal asymptote of the *A*/c_i_ curve. Stomatal limitation was estimated at a CO_2_ concentration of 400 μmol mol^−1^ from fitted C_4_ curves using the equation:

$$l=\frac{A_{0}-A_{\mathrm{sat}}}{A_{0}},$$

where *A*_0_ is the rate of photosynthesis that would occur at infinite stomatal conductance (Farquhar & Sharkey, 1982).

### 2.3 Quantifying Rubisco content, activation state and activity

Rubisco activation state was determined from measurements of initial and total (or fully activated) Rubisco catalytic sites (Butz & Sharkey, 1989; Galmés et al., 2011; Ruuska et al., 1998; von Caemmerer et al., 2005). Catalytic sites were measured by the binding of carboxypentitol‐1,5‐bisphosphate (^14^C‐CPBP) using size exclusion chromatography following Kubien, Brown, and Kane (2011), with modifications to quantify initial sites as described in Butz and Sharkey (1989). Purified C_3_ Rubisco was inactivated and measured to ensure the protocol did not return artificially high estimates of activation states (Figure S1). Leaf samples were ground in an ice‐cooled Tenbroeck glass homogenizer, containing an extraction buffer of 50 mM EPPS‐NaOH, 1 mM EDTA, 5 mM MgCl_2_, 1% PVPP, 5 mM DTT, and 1% protease inhibitor cocktail (Sigma‐Aldrich P9599) (pH = 8.0). The extraction was immediately placed in a centrifuge (Centrifuge 5,415 D, Eppendorf) at maximum speed (13,200 rpm) for 30 sec to pellet the cell debris, and initial reactions were initiated within 1 min of extraction. To quantify the initial amount of active Rubisco catalytic sites, 100 μl of supernatant was aliquoted into a tube with 22 μM ^14^C‐CPBP (2.96 × 10^4^ DPM nmol^−1^, prepared as described by Kubien et al., 2011) and placed on ice for 30 min. Then, 1.5 mM unlabelled CPBP and 100 μl of activation buffer (50 mM EPPS‐NaOH pH 8, 1 mM EDTA, 20 mM MgCl_2_, 30 mM NaHCO_3_) were added and incubated at room temperature for 20 min to allow any ^14^C‐CPBP originally bound to uncarbamylated sites to be replaced by the excess of unlabelled CPBP (Butz & Sharkey, 1989; Pierce, Tolbert, & Barker, 1980). After isotope exchange, samples underwent size exclusion chromatography, as described below. To quantify total Rubisco content in the sample, 100 μl of supernatant was aliquoted into a tube containing activation buffer and was incubated at room temperature for 20 min. This activated sample was then incubated with 3 mM ^14^C‐CPBP at room temperature for 30 min.

Rubisco‐bound ^14^C in initial and total samples was separated from unbound ^14^C via size exclusion chromatography using 0.7 × 30 cm columns packed with Sephadex G‐50 (Sigma‐Aldrich G5050), equilibrated with 20 mM EPPS, 75 mM NaCl (pH 8.0). Aliquots were measured by liquid scintillation counting (Packard Tri‐Carb 1900 TR, Canberra Packard Instruments Co., Downers Grove, IL). Activation state was calculated as the ratio of the number of initial Rubisco active catalytic sites to fully activated Rubisco catalytic sites (Butz & Sharkey, 1989). A Bradford assay (BioRad 5,000,001) was used to determine total soluble protein in the supernatant.

Rubisco activity was determined at 25°C spectrophotometrically via the rate of NADH oxidation at 340 nm using a diode‐array spectrophotometer (Agilent Cary 60 UV/Vis) (Kubien et al., 2011; Sharwood, Sonawane, Ghannoum, & Whitney, 2016). Paired leaf samples to those used to determine Rubisco content and activation state were extracted as described above, samples were activated in 1 ml cuvettes containing assay buffer (100 mM EPPS‐NaOH, pH 8.0, 10 mM MgCl2, 0.2 mM NADH, 20 mM NaHCO3, 1 mM ATP, pH 7.0, 5 mM phosphocreatine, pH 7.0, and 4% [v/v] coupling enzymes) for 15 minutes, and reactions initiated with the addition of 0.4 mM RuBP. RuBP for these assays was synthesized and purified as described by Kane, Wilkin, Portis, and Andrews (1998).

### 2.4 Quantification of ROS scavenging metabolites

At midday on June 23, 2018 and July 6, 2018 leaf samples for measuring phenolic content, ascorbate content, glutathione content, sugar content and chlorophyll content were taken on the marked eighth leaf. These samples were taken with a cork borer and immediately frozen in liquid N. The antioxidants pools were measured to gain insight in the antioxidant capacity of the plant. Chlorophyll and foliar glucose, fructose and sucrose were measured as described in Yendrek, Leisner, and Ainsworth (2013). A leaf sample of 1.34 cm^2^ was processed for total foliar phenolic content as described in Ainsworth and Gillespie (2007). In short, samples were extracted in 95% methanol and incubated in the dark at room temperature for 48 hr. The samples were then incubated with 10% Folin–Ciocalteu solution and 700 mM Na_2_CO_3_ at room temperature for 2 hr. Finally, absorbance was measured at 765 nm and compared to a standard curve of gallic acid. A GSH/GSSG‐Glo Assay kit (Promega Corporation, Madison, WI) was used to quantify glutathione content using a luminescence reaction following the manufacturer's protocol. Ten milligram of leaf tissue was mixed with 1× phosphate‐buffered saline with 2 mM EDTA (pH 8.0). Total glutathione content was measured by detecting a luciferase signal, which was proportional to glutathione content. Total and reduced ascorbate were measured using the methods of Gillespie and Ainsworth (2007) using a leaf sample of 1.9 cm^2^.

Samples for specific leaf area (SLA) were taken with a cork borer (1.9 cm^2^) and placed into a coin envelope. SLA samples were dried at 60°C for 10 days until they reached a constant mass. They were weighed and then ground to a fine powder. A small amount of each sample was weighed into a tin capsule for C and N analysis. An elemental analyzer (Costech 4010CHNSO Analyzer, Costech Analytical Technologies Inc. Valencia, CA) was used to measure C and N content. Acetanilide and apple leaves (National Institute of Science and Technology, Gaithersburg, MD) were used as standards.

### 2.5 Final harvest

On September 21, 2018, when plants had reached maturity, ears were harvested from eight plants per genotype per ambient and elevated O_3_ plot. Ears were dried in a drying oven at ~50°C until dry. Kernel mass per plant (yield) was calculated by dividing the total kernel mass by the number of plants harvested. A randomly selected sample of 100 kernels was taken from each ambient and elevated O_3_ genotype sub‐plot and weighed to estimate individual kernel mass.

### 2.6 Statistical analysis of physiological and biochemical traits

The field design was a random complete block design (*n* = 4). The full model for this experiment included fixed effects for time point, genotype, treatment, their interactions and a random effect for block (*n* = 4). For quantification of Rubisco, only three of the four blocks were analysed. For all phenotypes, PROC UNIVARIATE (SAS, Version 9.4, Cary, NC) was used to test that data were normally distributed. Including both time points together resulted in a bimodal distribution of data. Therefore, traits were analysed by time point using a two‐way ANOVA with genotype, treatment and the interaction as fixed terms and block as a random term (Proc Mixed; SAS, Version 9.4, Cary, NC). Least squares means were estimated and used to compare means in ambient and elevated O_3_ within a genotype using pairwise comparisons.

## 3 RESULTS

### 3.1 In vivo and in vitro photosynthetic response of maize to elevated O_3_

**FIGURE 2:**
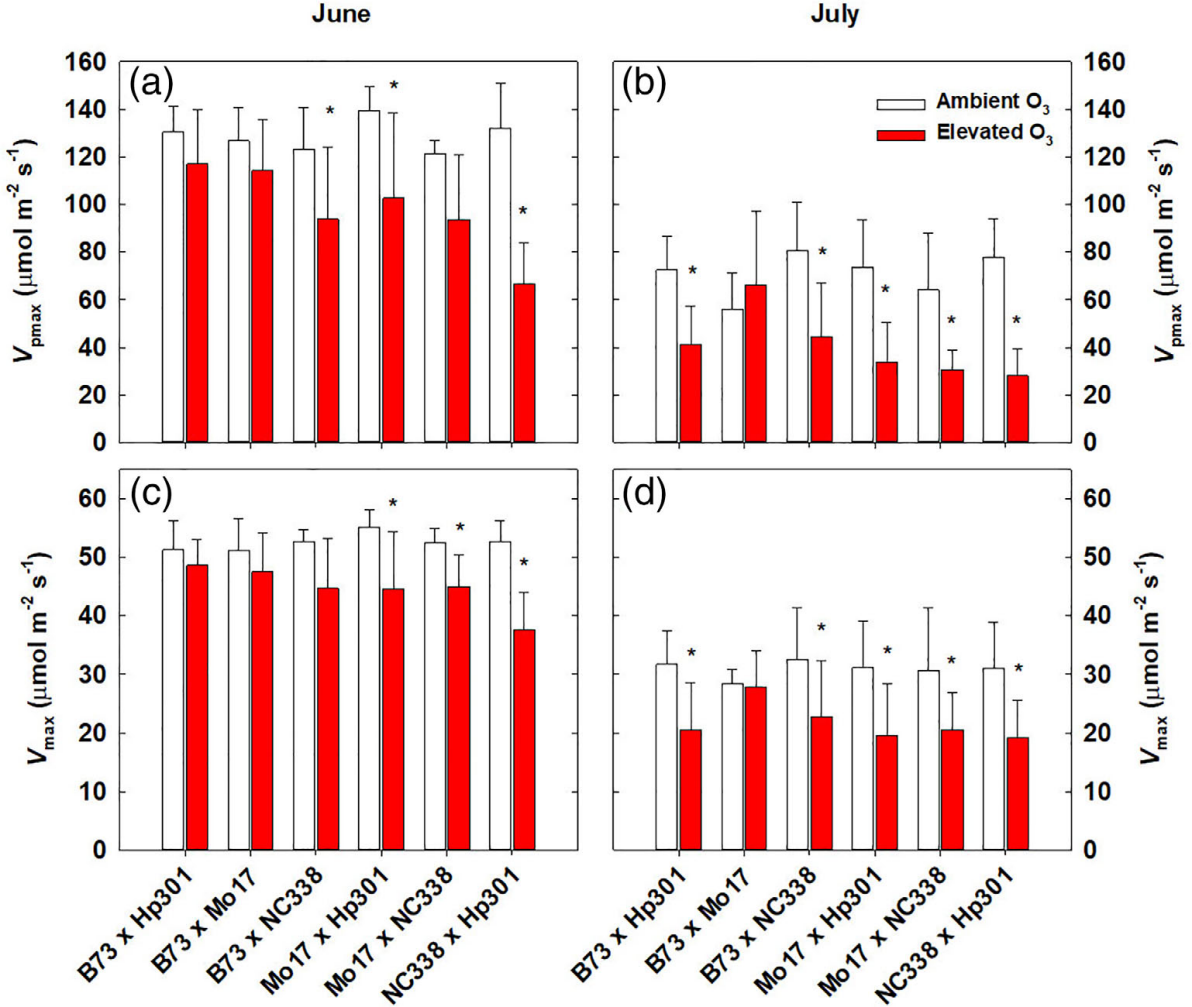
In vivo measurements of *V*_*pmax*_ (a, b) and *V*_*max*_ (c, d) in genotypes grown in ambient and elevated O_3_ in June and July. Error bars represent standard deviation. *Significant (*p* < .05) pairwise comparison between treatments within each genotype in each time point

Prolonged exposure to elevated O_3_ decreased photosynthetic capacity in most of the maize hybrid lines (Figure 2a–d). There was a significant decrease in elevated O_3_ in *V*_*pmax*_ and *V*_*max*_ estimated from gas exchange in June and July (Table S1). Pairwise comparisons within each genotype demonstrated that three of the six genotypes had reduced *V*_*pmax*_ and *V*_*max*_ in elevated O_3_ in June (Figure 2a,c). However, in July there were large reductions in *V*_*pmax*_ and *V*_*max*_ in elevated O_3_ for all the genotypes except B73 × Mo17, which showed no difference in *V*_*pmax*_ and *V*_*max*_ in ambient and elevated O_3_ (Figure 2b,d).

**FIGURE 3:**
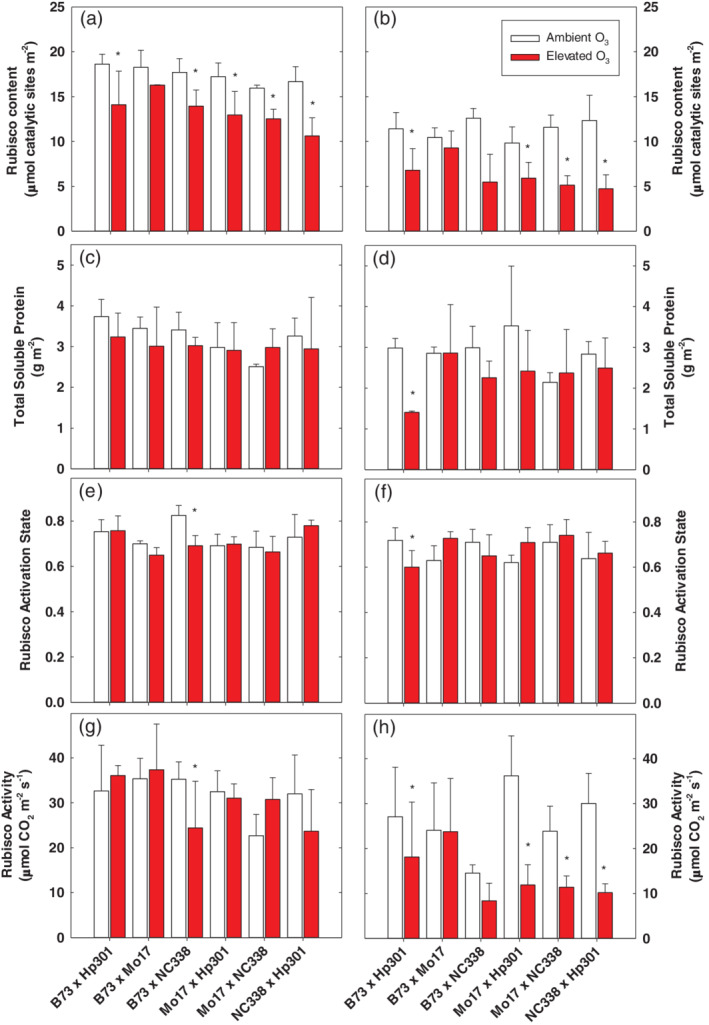
Rubisco content (a and b), Soluble protein (c and d), activation state (e and f), and in vitro Rubisco activity (g and h) across the six genotypes in ambient and elevated O_3_ in June and July. Error bars represent standard deviation. *Significant (*p* < .05) pairwise comparison between treatments within each genotype in each time point

Rubisco content measured in vitro was substantially reduced in elevated O_3_ in June in all genotypes (Figure 3a), and July in all genotypes except B73 × Mo17 (Figure 3b; *p* < .0001) (Table S1). Changes in Rubisco content were generally not reflected in reductions in total soluble protein in elevated O_3_ in June or July (Figure 3c,d), indicating that reductions in Rubisco content were independent of global protein down‐regulation. Interestingly, the activation state of Rubisco did not change between June and July (Figure 3e,f) and was not significantly affected by elevated O_3_ (Table S1). Measurements of in vivo Rubisco activity were correlated with Rubisco content (Figure S2), although there was no significant effect of O_3_ on Rubisco activity in June (Figure 3g) when content was significantly lower (Figure 3a). In July, B73 × Mo17 showed no significant decrease in Rubisco content or activity in contrast to the other hybrid lines (Figure 3h).

**FIGURE 4:**
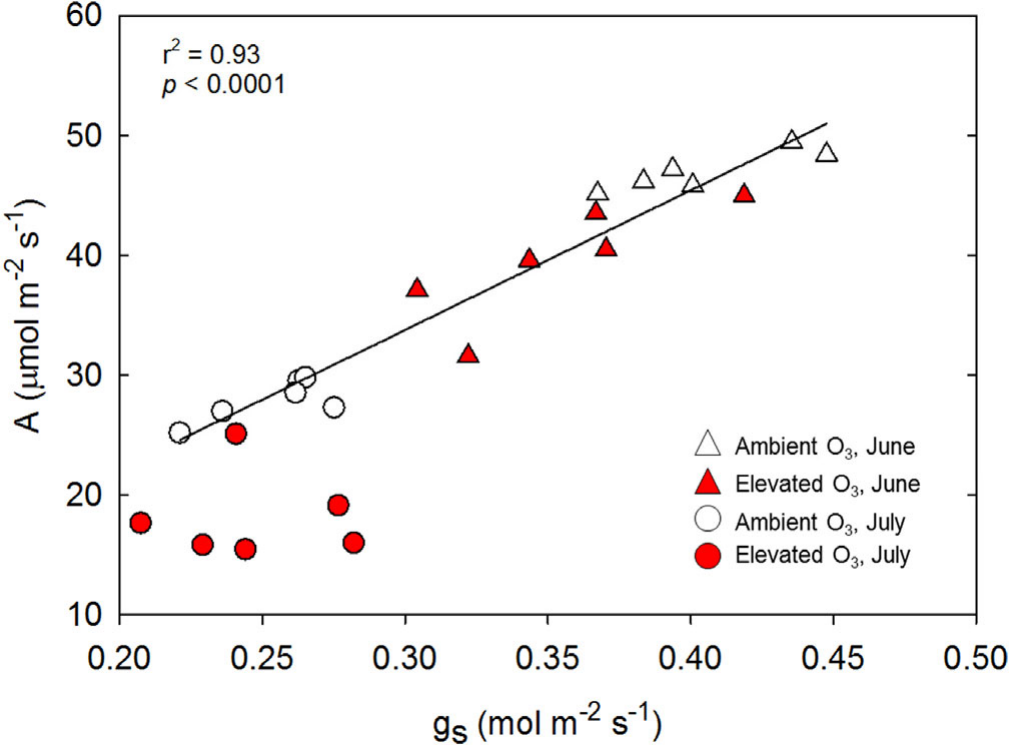
The relationship between light‐saturated photosynthetic CO_2_ assimilation rate (*A*) and stomatal conductance (*g*_*s*_) measured in ambient (open symbols) and elevated O_3_ (red symbols) in June (triangles) and July (hexagons). Prolonged exposure to elevated O_3_ disrupts the linear relationship between *A* and g_s_

Reduced photosynthetic capacity resulted in lower net photosynthetic rates (*A*) in elevated O_3_, with rates decreased by 16% in June and by 34% in July (Table 1). Although no significant genotype–treatment interaction was detected in the statistical model (Table S2), the magnitude of the response of *A* to elevated O_3_ ranged from a 7% decrease in elevated O_3_ in B73 × Mo17 to a 40% decrease in NC338 × Hp301 in June. Stomatal conductance (g_s_) was also significantly lower in elevated O_3_ in June, but not in July (Tables 1 and S2). Thus, prolonged exposure to elevated O_3_ in hybrid maize altered the linear relationship between *A* and g_s_ (Figure 4). Intercellular [CO_2_] (c_i_) was also significantly greater in elevated O_3_, especially in July (Table 1). NC338 × Hp301 showed the greatest change in c_i_ in both June and July, whereas B73 × Mo17 showed no change in g_s_ or c_i_ under elevated O_3_ compared to ambient O_3_ in July (Table 1). Stomatal limitation to photosynthesis (*l*) did not consistently respond to elevated O_3_, and tended to be slightly higher in elevated O_3_ in June and lower in elevated O_3_ in July (Tables 1 and S2). Pairwise comparisons showed that only genotype B73 × Mo17 showed a significant reduction in stomatal limitation in elevated O_3_ in July (Table 1).

**TABLE 1:** Least squared means for gas exchange traits in ambient and elevated O_3_ in June and July

		*A* (μmol m^−2^ s^−1^)		*g*_*s*_ (Mol m^−2^ s^−1^)		*c*_*i*_ (μmol Mol^−1^)		Stomatal limitation	
	Time point	Ambient	Elevated	Ambient	Elevated	Ambient	Elevated	Ambient	Elevated
B73 × Hp301	June	48.4 ± 3.0	45.0 ± 5.4	0.45 ± 0.04	0.42 ± 0.05	144.7 ± 10.5	151.2 ± 12.6	0.06 ± 0.02	0.07 ± 0.03
B73 × Mo17	June	47.2 ± 2.1	43.5 ± 6.0	0.39 ± 0.02	0.37 ± 0.06	128.3 ± 6.9	136.1 ± 12.5	0.09 ± 0.03	0.09 ± 0.03
B73 × NC338	June	45.9 ± 2.5	40.5 ± 8.1	0.40 ± 0.05	0.37 ± 0.07	137.6 ± 14.4	152.6 ± 26.3	0.09 ± 0.04	0.12 ± 0.08
Mo17 × Hp301	June	**49.5 ± 1.3**	**39.6 ± 10.1** ^*^	**0.44 ± 0.04**	**0.34 ± 0.07** ^*^	133.6 ± 9.2	148.8 ± 28.1	0.08 ± 0.03	0.11 ± 0.04
Mo17 × NC338	June	**45.2 ± 2.5**	**37.1 ± 8.6** ^*^	0.37 ± 0.04	0.30 ± 0.09	124.9 ± 7.8	136.2 ± 11.1	0.12 ± 0.06	0.12 ± 0.06
NC338 × Hp301	June	**46.2 ± 1.6**	**31.6 ± 6.4** ^*^	0.38 ± 0.04	0.32 ± 0.07	**128.3 ± 13.4**	**185.2 ± 18.3** ^*^	0.10 ± 0.03	0.13 ± 0.06
B73 × Hp301	July	**29.6 ± 3.6**	**17.7 ± 5.5** ^*^	0.26 ± 0.01	0.21 ± 0.07	**167.3 ± 20.6**	**224.3 ± 21.2** ^*^	0.07 ± 0.04	0.06 ± 0.04
B73 × Mo17	July	25.2 ± 2.1	25.1 ± 6.8	0.22 ± 0.02	0.24 ± 0.04	168.9 ± 8.1	184.8 ± 41.4	**0.10 ± 0.06**	**0.06 ± 0.02** ^*^
B73 × NC338	July	**29.8 ± 7.4**	**19.2 ± 8.8** ^*^	0.27 ± 0.06	0.28 ± 0.06	**166.8 ± 15.8**	**249.7 ± 52.4** ^*^	0.06 ± 0.02	0.05 ± 0.03
Mo17 × Hp301	July	**27.3 ± 4.0**	**15.9 ± 6.8** ^*^	0.28 ± 0.05	0.23 ± 0.06	**186.4 ± 29.3**	**257.2 ± 41.6** ^*^	0.07 ± 0.03	0.06 ± 0.02
Mo17 × NC338	July	**27.0 ± 8.1**	**16.0 ± 4.9** ^*^	0.24 ± 0.06	0.28 ± 0.03	**167.9 ± 20.7**	**273.5 ± 27.4** ^*^	0.10 ± 0.06	0.07 ± 0.03
NC338 × Hp301	July	**28.6 ± 5.4**	**15.5 ± 4.8** ^*^	0.26 ± 0.05	0.24 ± 0.08	**173.9 ± 14.7**	**264.5 ± 17.4** ^*^	0.06 ± 0.04	0.08 ± 0.02

*Note:* Asterisks (*) and bold font represent significant pairwise comparison within each genotype for each time point (*p* < .05).

### 3.2 Biochemical responses of hybrid maize to elevated O_3_

**FIGURE 5:**
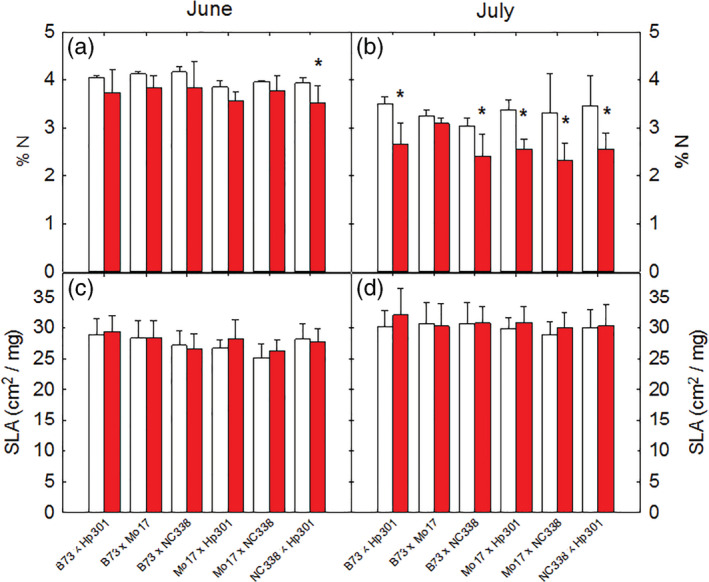
Percent nitrogen (% N) and specific leaf area (SLA) measured in ambient and elevated O_3_ in June (a) and July (b). Error bars show standard deviation. *Significant (*p* < .05) pairwise comparison between treatments within each genotype in each time point

Overall, percent nitrogen (%N) decreased in elevated O_3_ in both June and July (Figure 5a,b; Table S3) with a much greater reduction in July. B73 × Mo17 showed no change in %N in elevated O_3_ in either June or July, consistent with dampened O_3_ response of other physiological traits. Specific leaf area was not affected by growth at elevated O_3_ in any of the hybrids (Figure 5c,d, Table S3).

Chlorophyll a and b were decreased in elevated O_3_ (Tables 2 and S4). All genotypes except B73 × Hp301 and Mo17 × Hp301 showed significant reductions in chlorophyll a and b in June. In July, all genotypes besides B73 × Mo17 had substantial decreases in chlorophyll a and b in elevated O_3_. The ratio of chlorophyll a to b was unchanged in June in elevated O_3,_ but increased in elevated O_3_ in July for most genotypes (Table 2). Carotenoids were not impacted by elevated O_3_ in June and were too low to be detected in July (Table S4).

**TABLE 2:** Least squared means for chlorophyll content across all six genotypes in ambient and elevated O_3_ in June and July

		Chlorophyll a (μg cm^−2^)		Chlorophyll b (μg cm^−2^)		Carotenoids (μg cm^−2^)		Chl a/Chl b	
	Time point	Ambient	Elevated	Ambient	Elevated	Ambient	Elevated	Ambient	Elevated
B73 × Hp301	June	26.4 ± 2.5	23.8 ± 3.2	7.5 ± 0.6	6.7 ± 1.1	1.89 ± 0.27	1.92 ± 0.23	3.53 ± 0.14	3.55 ± 0.17
B73 × Mo17	June	**29.5 ± 1.6**	**25.3 ± 3.3** ^*^	**8.2 ± 0.5**	**7.1 ± 1.1** ^*^	2.20 ± 0.13	1.98 ± 0.16	3.6 ± 0.09	3.58 ± 0.18
B73 × NC338	June	**27.5 ± 2.7**	**22.0 ± 3.6** ^*^	**8.0 ± 1.2**	**6.1 ± 1.0** ^*^	1.78 ± 0.20	1.69 ± 0.34	3.49 ± 0.23	3.59 ± 0.24
Mo17 × Hp301	June	22.8 ± 1.8	22.6 ± 1.9	6.6 ± 0.5	6.6 ± 0.7	1.77 ± 0.31	1.69 ± 0.24	3.46 ± 0.22	3.44 ± 0.19
Mo17 × NC338	June	**25.3 ± 2.6**	**21.7 ± 2.5** ^*^	**7.3 ± 0.5**	**6.1 ± 1.0** ^*^	1.58 ± 0.23	1.68 ± 0.19	3.45 ± 0.14	3.61 ± 0.24
NC338 × Hp301	June	**21.2 ± 1.0**	**17.8 ± 2.7** ^*^	5.7 ± 0.3	4.9 ± 0.6	1.58 ± 0.20	1.46 ± 0.24	3.71 ± 0.26	3.59 ± 0.27
B73 × Hp301	July	**15.3 ± 2.2**	**10.9 ± 2.0** ^*^	**6.6 ± 0.8**	**4.3 ± 1.0** ^*^	—	—	**2.33 ± 0.14**	**2.6 ± 0.24** ^*^
B73 × Mo17	July	17.4 ± 2.5	16.6 ± 3.2	7.1 ± 1.0	6.3 ± 1.0	—	—	**2.46 ± 0.18**	**2.64 ± 0.15** ^*^
B73 × NC338	July	**16.0 ± 2.4**	**11.5 ± 2.9** ^*^	**6.9 ± 1.0**	**4.4 ± 1.3** ^*^	—	—	**2.31 ± 0.15**	**2.62 ± 0.18** ^*^
Mo17 × Hp301	July	**13.6 ± 2.2**	**10.7 ± 2.8** ^*^	**6.2 ± 1.1**	**4.3 ± 1.2** ^*^	—	—	2.22 ± 0.14	2.50 ± 0.15
Mo17 × NC338	July	**16.3 ± 2.3**	**11.9 ± 2.4** ^*^	**7.1 ± 0.8**	**4.9 ± 1.1** ^*^	—	—	2.30 ± 0.19	2.46 ± 0.19
NC338 × Hp301	July	**14.6 ± 3.1**	**9.6 ± 2.4** ^*^	**6.2 ± 1.1**	**3.7 ± 1.1** ^*^	—	—	**2.35 ± 0.20**	**2.60 ± 0.22** ^*^

*Note:* Asterisks (*) and bold font represent significant pairwise comparison within each genotype for each time point (*p* < .05).

Phenolic, ascorbate and glutathione contents were measured in all six genotypes in June and July to determine if there were genotypic differences in antioxidant responses to elevated O_3_ (Table 3). Across all hybrids, there was a significant O_3_ effect on phenolic content with increased levels in elevated O_3_ in June (*p* < .05), but there was no effect of O_3_ on phenolic compounds in July (Table S5). However, there were no significant pairwise comparisons in phenolic content in elevated O_3_ within hybrid lines in either June or July. A similar pattern was found for ascorbate. Total ascorbate was not changed in elevated O_3_ in June or July, but the redox state of ascorbate was generally increased under elevated O_3_ (Table S5). Three hybrids showed increases in the redox state of ascorbate in elevated O_3_ based on pair‐wise comparisons. There were no significant pairwise differences within the hybrids between ambient and elevated O_3_ for total glutathione content in June. In July, genotypes B73 × Hp301 and NC338 × Hp301 had reductions of total foliar glutathione in elevated O_3_ without any change in redox status of the glutathione pool.

**TABLE 3:** Least squared means for antioxidants for each genotype in ambient and elevated O_3_ in June and July

		Phenolics (μmol cm^−2^)		Total ascorbate (nmol cm^−2^)		%reduced (ascorbate)		Total glutathione (nmol cm^−2^)		%reduced (glutathione)	
	Time point	Ambient	Elevated	Ambient	Elevated	Ambient	Elevated	Ambient	Elevated	Ambient	Elevated
B73 × Hp301	June	0.49 ± 0.1	0.56 ± 0.09	114.4 ± 16.3	135.2 ± 21.8	49.8 ± 6.1	47.2 ± 7.5	3.99 ± 0.46	4.75 ± 0.34	71.7 ± 4.1	73.5 ± 8.2
B73 × Mo17	June	0.49 ± 0.1	0.56 ± 0.11	145.8 ± 33.2	174.3 ± 16.7	55.2 ± 6.5	59.7 ± 9.1	2.73 ± 1.05	2.49 ± 0.75	75.2 ± 6.3	74.6 ± 8.1
B73 × NC338	June	0.54 ± 0.09	0.62 ± 0.12	143.3 ± 37.0	158.2 ± 42.5	52.5 ± 6.3	60.2 ± 4.2	2.85 ± 0.37	3.67 ± 1.10	69.4 ± 4.1	73.3 ± 5.8
Mo17 × Hp301	June	0.49 ± 0.07	0.53 ± 0.08	132.2 ± 31.0	133.4 ± 22.7	51.4 ± 7.1	60.0 ± 9.9	4.29 ± 0.53	5.35 ± 0.87	74.0 ± 3.2	71.6 ± 2.1
Mo17 × NC338	June	0.58 ± 0.09	0.53 ± 0.11	141.8 ± 10.5	128.5 ± 16.8	63.5 ± 6.4	60.3 ± 5.5	4.25 ± 0.58	3.75 ± 1.01	74.0 ± 4.0	75.6 ± 2.8
NC338 × Hp301	June	0.51 ± 0.08	0.60 ± 0.16	151.2 ± 33.5	157.6 ± 47.5	51.7 ± 2.6	55.4 ± 11.8	5.42 ± 1.01	6.21 ± 2.63	**77.4 ± 2.3**	**68.1 ± 8.4** ^*^
B73 × Hp301	July	0.38 ± 0.06	0.37 ± 0.06	128.4 ± 32.6	153.8 ± 14.4	35.7 ± 8.2	43.4 ± 3.8	**2.17 ± 0.36**	**1.33 ± 0.69** ^*^	80.2 ± 6.5	78.1 ± 14.2
B73 × Mo17	July	0.35 ± 0.05	0.36 ± 0.06	137.1 ± 16.7	146.5 ± 8.4	39.1 ± 5.1	39.0 ± 5.4	1.44 ± 0.17	1.38 ± 0.55	83.2 ± 5.6	80.0 ± 11.4
B73 × NC338	July	0.38 ± 0.06	0.41 ± 0.07	148.5 ± 20.1	148.9 ± 14.9	**40.1 ± 5.8**	**53.1 ± 9.6** ^*^	1.53 ± 0.54	1.13 ± 0.63	79.9 ± 9.2	88.2 ± 4.6
Mo17 × Hp301	July	0.33 ± 0.04	0.33 ± 0.05	122.9 ± 29.6	111.3 ± 11.5	33.6 ± 8.0	40.6 ± 15.4	2.15 ± 0.31	1.72 ± 0.42	85.0 ± 5.9	80.4 ± 3.4
Mo17 × NC338	July	0.33 ± 0.07	0.38 ± 0.09	149.1 ± 14.2	141.2 ± 37.2	**39.2 ± 6.3**	**49.6 ± 6.7** ^*^	1.84 ± 0.37	1.37 ± 0.21	76.1 ± 3.3	85.0 ± 3.7
NC338 × Hp301	July	0.38 ± 0.05	0.41 ± 0.07	144.1 ± 23.9	156.8 ± 30.8	**41.0 ± 2.5**	**46.3 ± 7.0** ^*^	**2.75 ± 0.58**	**1.29 ± 0.39** ^*^	73.2 ± 6.2	82.7 ± 7.8

*Note:* Asterisks (*) and bold font represent significant pairwise comparison within each genotype for each time point (*p* < .05).

There were no changes in foliar glucose or sucrose in ambient and elevated O_3_ in June (Tables 4 and S6). Only B73 × Hp301 showed significant decreases in foliar glucose and sucrose under elevated O_3_ in July (Table 4). There was a consistent increase in foliar fructose under elevated O_3_ in June, but the response was inconsistent in July (Table S6). Similarly, glucose showed no consistent pattern in June, but decreased in elevated O_3_ across all hybrids in July (Table S6). Sucrose remained unchanged between ambient and elevated O_3_ in both June and July.

**TABLE 4:** Least squared means for carbohydrates in ambient and elevated O_3_ in June and July

		Glucose (nmol cm^−2^)		Fructose (nmol cm^−2^)		Sucrose(nmol cm^−2^)	
	Time point	Ambient	Elevated	Ambient	Elevated	Ambient	Elevated
B73 × Hp301	June	147.8 ± 41.9	158.1 ± 67.7	80.7 ± 47.0	114.8 ± 69.7	954.3 ± 292.7	1,092.0 ± 285.0
B73 × Mo17	June	172.3 ± 62.1	167.9 ± 90.9	62.0 ± 26.0	108.1 ± 55	1,129 ± 189.7	1,246.1 ± 281.9
B73 × NC338	June	143.4 ± 38.8	147.3 ± 50.3	76.0 ± 30.4	117.7 ± 69.4	1,057.5 ± 277.4	1,216.0 ± 315.7
Mo17 × Hp301	June	105.9 ± 40.0	148.8 ± 53.5	52.2 ± 32.9	73.7 ± 55.7	1,036.3 ± 344.7	954.2 ± 250.3
Mo17 × NC338	June	146.8 ± 47.9	175.3 ± 74.6	90.0 ± 34.2	114.1 ± 61.2	1,112.7 ± 298.5	1,089.2 ± 336.7
NC338 × Hp301	June	128.2 ± 44.2	157.4 ± 47.1	100.3 ± 27.3	142.8 ± 67.2	875.0 ± 260.2	957.1 ± 277.9
B73 × Hp301	July	**125.1 ± 37.9**	**82.3 ± 38.7** ^*^	**80.4 ± 45.6**	**42.5 ± 26.8** ^*^	913.7 ± 330.8	798.1 ± 240.1
B73 × Mo17	July	133.0 ± 73.4	131.5 ± 61.3	71.4 ± 47.0	90.6 ± 47.9	1,106.2 ± 556.8	1,116.6 ± 419.6
B73 × NC338	July	113.5 ± 46.3	105.2 ± 35.9	56.9 ± 31.6	68.6 ± 38.2	964.3 ± 358.6	797.8 ± 330.9
Mo17 × Hp301	July	99.4 ± 47.3	73.4 ± 34.9	50.4 ± 33.6	32.3 ± 21.3	958.1 ± 808.2	719.9 ± 221.0
Mo17 × NC338	July	118.2 ± 46.5	114.4 ± 87.8	53.7 ± 21.0	43.0 ± 34.7	967.9 ± 363.0	816.0 ± 317.5
NC338 × Hp301	July	106.5 ± 39.8	82.9 ± 23.0	58.5 ± 26.0	59.1 ± 44.3	792.0 ± 337.0	725.1 ± 306.8

*Note:* Asterisks (*) and bold font represent significant pairwise comparison within each genotype for each time point (*p* < .05).

### 3.3 Correlation between *A*, N, Rubisco content and yield

**FIGURE 6:**
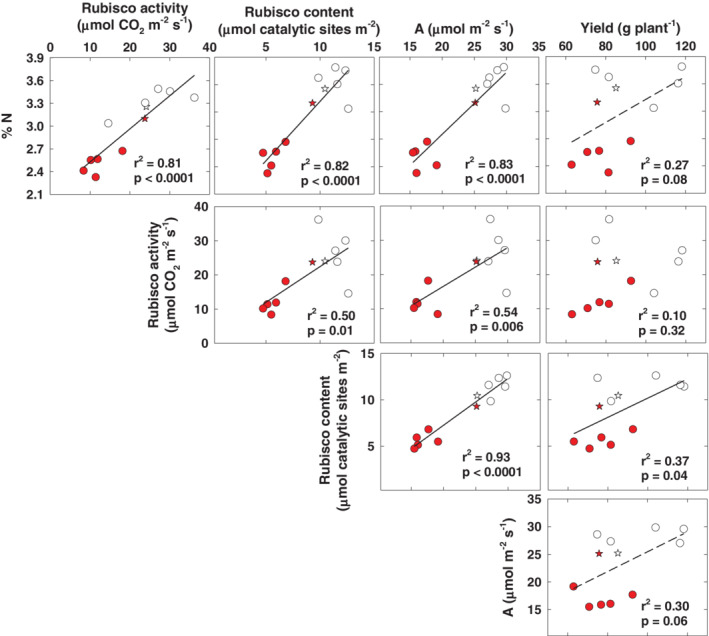
Correlations between % N, total Rubisco activation, net photosynthesis (*A*) measured in July and yield in ambient and elevated O_3_. White points indicate measurements in ambient O_3,_ and red points indicate measurements in elevated O_3_. The star symbols represent genotype B73 × Mo17

Exposure to elevated O_3_ significantly decreased yield and individual seed weight (Table S7). Foliar N was strongly correlated with *A* (Figure 6b) and Rubisco content (Figure 6a), and weakly correlated with seed yield (Figure 6c). Total Rubisco content was positively and significantly correlated with *A* and seed yield across O_3_ treatments (Figure 6d,e) and *A* was weakly correlated with seed yield, largely because the hybrids showed a range of yield values in ambient O_3_, but little variation in *A* (Figure 6e). B73 × Mo17, which maintained high %N and photosynthetic capacity, was more tolerant to O_3_ stress than the other hybrids (indicated by stars in Figure 6).

## 4 DISCUSSION

Current tropospheric O_3_ is estimated to cause yield losses in maize up to 15% (Mills, Sharps, et al., 2018c), which translates to losses up to $9 billion in the US (McGrath et al., 2015). In this study, we used FACE technology to examine the effects of long‐term exposure to elevated O_3_ on the photosynthetic capacity of maize hybrids. Growth at elevated O_3_ reduced photosynthetic capacity, measured both in vivo and in vitro, except for B73 × Mo17, which showed greater resistance to elevated O_3_ pollution than other maize hybrids. Based on previous experiments, investigating leaf‐level photosynthetic responses of maize to O_3_ (Choquette et al., 2019), we hypothesized that differences in antioxidant capacity and/or stomatal responses to elevated O_3_ would predict genetic variation in photosynthetic responses to elevated O_3_. However, we found no evidence for significant variation among genotypes in stomatal limitation responses to elevated O_3_, and little difference in the antioxidant response among the genotypes. Conversely, we found significant genetic variation in Rubisco activity responses to elevated O_3_, which was correlated with yield across diverse maize hybrids.

Antioxidant capacity in the apoplast provides the first line of defence against O_3_ and other ROS (Kangasjärvi, Jaspers, & Kollist, 2005) and is linked to tolerance to multiple environmental stresses (Scebba, Pucciarelli, Soldatini, & Ranieri, 2003). Phenolic molecules directly scavenge ROS (Grace & Logan, 2000), respond to stresses that impair photosynthesis (Koricheva, Larsson, Haukioja, & Keinanen, 1998) and increase under elevated O_3_ in a variety of species (Gillespie, Rogers, & Ainsworth, 2011; Kangasjärvi, Talvinen, Utriainen, & Karjalainen, 1994; Peltonen, Vapaavuori, & Julkunen‐Tiitto, 2005; Yendrek, Koester, & Ainsworth, 2015). Therefore, we hypothesized that antioxidant and phenolic compounds would vary among tolerant and sensitive maize lines. However, we did not find evidence for genotypic variation in antioxidant and phenolic responses to O_3_. While there was a significant effect of O_3_ on phenolic content across all genotypes in June (Table 3), older maize leaves measured in July had lower phenolic content and no response to elevated O_3_. A study of wheat and maize also showed decreased phenolic content over time with exposure to O_3_ (Li, Shi, & Chen, 2008). Antioxidants, glutathione and ascorbate, are important to ROS scavenging (Foyer & Noctor, 2005), and reduced glutathione donates an electron to dehydroascorbate, which regenerates oxidized ascorbate into reduced ascorbate (Kangasjärvi et al., 1994). Although there were genotypic differences in the pool sizes of total glutathione across both time points, the redox state was the same between ambient and elevated O_3_. The redox state of ascorbate generally increased in elevated O_3_ consistent with a study of juvenile maize grown under oxidative stress, which found no change in the redox state of glutathione and an increase in the redox state of ascorbate (Kingston‐Smith, Harbinson, & Foyer, 1999).

Previous studies of these maize genotypes identified variation in leaf‐level photosynthetic responses to O_3_, and it was hypothesized that variation in stomatal limitation may be associated (Choquette et al., 2019). However, contrary to our hypothesis, there was no evidence for genetic variation in stomatal limitation to photosynthesis under O_3_ stress. Stomatal limitation tended to be slightly higher in elevated O_3_ in June across all genotypes, but pairwise comparisons among genotypes did not identify any significant differences between ambient and elevated O_3_. Instead, gas exchange measurements revealed that both PEP carboxylase activity (*V*_*pmax*_) and maximum photosynthetic rates (*V*_*max*_) were reduced by exposure to elevated O_3_ (Figure 2), and there was significant genetic variation in O_3_ response of *V*_*pmax*_ in older leaves. in vitro experiments also revealed that both Rubisco content and activity were reduced by elevated O_3_, and there was genetic variation in response, particularly in July after prolonged exposure to the pollutant (Figure 3)_._ The total Rubisco content, we report in this study, is similar to Rubisco content in maize reported by Salesse‐Smith et al. (2018), and we found that Rubisco content and activity decreased with leaf age, as previously reported (Sharwood et al., 2016). Other studies have reported decreases in initial and total Rubisco activity in many different species under elevated O_3_ (Brendley & Pell, 1998; Dann & Pell, 1989; Eckardt & Pell, 1994; Galant, Koester, Ainsworth, Hicks, & Jez, 2012; Pell & Pearson, 1983; Pelloux, Jolivet, Fontaine, Banvoy, & Dizengremel, 2001; Reid, Fiscus, & Burkey, 1998). In an experiment on juvenile maize, PEP carboxylase and Rubisco content and activity decreased with increasing O_3_ exposure (Leitao, Bethenod, & Biolley, 2007), which is consistent with our results for sensitive hybrids. A meta‐analysis of Rubisco content and activity also found that O_3_ reduced Rubisco concentration (Galmés, Aranjuelo, Medrano, & Flexas, 2013), possibly because of reduced synthesis of Rubisco messenger RNA (Heath, 2008) or enhanced degradation of Rubisco (Eckardt & Pell, 1994). It has been shown that ROS can accelerate Rubisco degradation in chloroplasts (Feller, Anders, & Mae, 2008), which is consistent with decreased Rubisco, but not overall soluble protein content as found in this study (Figure 3). Rubisco can also be regulated through redox potential (Huffaker, 1982) and has sulfhydryl groups that become oxidized and signal degradation under stress conditions and nutrient deficits (Garcia‐Ferris & Moreno, 1993; Garcia‐Ferris & Moreno, 1994; Pell & Pearson, 1983). It seems likely that chronic exposure to O_3_ in maize overwhelmed the detoxification potential of the cells, resulting in signalling cascades that triggered degradation of Rubisco enzymes, as has been postulated for other C_3_ species (Goumenaki et al., 2010).

Rubisco carboxylation can be inhibited by sugar phosphates and Rubisco activase is important for removing the inhibitors from catalytic sites in an ATP‐dependent manner. Rubisco activase restores Rubisco from an inactive to active conformation (Portis Jr., 2003) and is imperative to maintain photosynthesis in C_4_ plants, even with carbon concentration mechanisms (von Caemmerer et al., 2005). In this study, activation state of Rubisco, reflecting Rubisco activase activity, was not affected by elevated O_3_ and did not contribute to a loss in photosynthetic capacity in elevated O_3_ (Figure 3). A previous study in maize showed that Rubisco activase transcript levels did not change with O_3_ exposure in the fifth and 10th leaf (Leitao, Maoret, & Biolley, 2007), consistent with our findings. Although a study in *Pinus halepensis* M. demonstrated a small decrease in Rubisco activase concentration after exposure to O_3_, carbamylation of Rubisco remained unchanged (Pelloux et al., 2001). Our results support these previous experiments and extend the findings that activation of Rubisco by Rubisco activase was not affected by elevated O_3_ in maize.

In this study, maize Rubisco activation state ranged from 65 to 82% in June and 60–75% in July, consistent with other estimates of activation state in maize (Carmo‐Silva et al., 2010; Sharwood et al., 2016). In C_4_ plants, total Rubisco content is lower than in C_3_ species, yet activation state in C_4_ maize was similar or lower than in C_3_ species (Perdomo, Capo‐Bauca, Carmo‐Silva, & Galmes, 2017; Sharwood et al., 2016). In other studies, Rubisco activation state in C_4_ species was reported as low as 45–55% (Carmo‐Silva et al., 2010; von Caemmerer et al., 2005). It is generally believed that Rubisco carboxylation of CO_2_ is the ultimate limitation in C_4_ species (Edwards, Furbank, Hatch, & Osmond, 2001; von Caemmerer, Millgate, Farquhar, & Furbank, 1997), and over‐expression of Rubisco content in maize resulted in increased plant height and biomass (Salesse‐Smith et al., 2018). This begs the question, why would the activation state of Rubisco in C_4_ species be the same or lower than C_3_ species? It is possible that the carbon concentrating mechanism of Kranz leaf anatomy of C_4_ species might play a role as the CO_2_ concentration inside the bundle sheath is 10‐fold higher than the atmosphere (Furbank & Hatch, 1987; von Caemmerer & Furbank, 2003). The carbon concentrating mechanism may make it feasible for C_4_ species to over‐invest in Rubisco by a negligible amount in terms of N storage and maintain low‐levels of inactivated Rubisco. It is interesting that under different oxidative stress conditions and different leaf ages, the activation state of maize Rubisco remained relatively constant (Figure 3e,f).

Leaf N content was strongly correlated with Rubisco activity, Rubisco content and *A* in maize leaves exposed to elevated O_3_, which is predicted as Rubisco content and activity control net carbon assimilation and C_4_ plants allocate 5–10% of leaf N to Rubisco (Figure 6; Ghannoum et al., 2005; Sharwood et al., 2016). We also found that Rubisco content measured in a mature leaf in July was correlated to yield in maize lines exposed to elevated O_3_ (Figure 6). Previous work has demonstrated that Rubisco is an important storage protein for N, sulfur and carbon skeletons (Liu, Ren, White, Cong, & Lu, 2018; Sage & Pearcy, 1987), and is crucial for remobilization of N to seeds (Feller et al., 2008; Millard & Grelet, 2010). The fact that the tolerant hybrid B73 × Mo17 did not show significant reductions in Rubisco content in July in contrast to other hybrids supports the notion that acceleration of senescence is a key determinant of productivity responses to O_3_. O_3_ is found to trigger the expression of genes involved in senescence in plants (Lim, Kim, & Nam, 2007; Miller, Arteca, & Pell, 1999), and when a leaf undergoes senescence, many nutrients, such as nitrogen, phosphorus and metals, are recycled in the plants and nutrient‐rich molecules are degraded (Lim et al., 2007). Accelerated loss of photosynthetic capacity and leaf aging from elevated O_3_ has been demonstrated in many crops of wheat, rice, soybean and maize (Betzelberger et al., 2010; Emberson et al., 2018; Feng et al., 2011; Pang, Kobayashi, & Zhu, 2009), and is a target for improving crop tolerance to O_3_ (Yendrek, Erice, et al., 2017).

## 5 CONCLUSIONS

Ozone pollution is an important stressor on plants that reduces crop yields around the world. Ozone damage to plants is considered a threat to food security and could be exacerbated in a changing climate (Tai, Martin, & Heald, 2014). For maize, global O_3_ stress causes equivalent damage as nutrient deficiency, heat and drought stress (Mills, Sharps, et al., 2018c). Understanding how O_3_ impacts physiological and biochemical processes in plants is vital to combat O_3_ damage. Our study demonstrates that accelerated senescence from O_3_ pollution decreases photosynthetic capacity in vitro and in vivo but Rubisco activation state is unchanged in O_3_. Tolerance to O_3_ stress in B73 × Mo17 does not appear to be linked to antioxidant capacity or stomatal response, rather to maintenance of leaf photosynthetic protein content. In this work, we have reported declines in Rubisco content and activity in sensitive lines, but accompanying declines in leaf *V*_*pmax*_ imply that other enzymes involved in the C_4_ pathway may be similarly impacted by elevated [O_3_], and such characterization remains a target for future research. It would be interesting to study the mechanism controlling decreases in Rubisco content in the sensitive lines in elevated [O_3_], which could result from greater turnover of Rubisco, decreased mRNA abundance or problems with assembly resulting from damage or declines in chaperones and assembly factors. More detailed transcriptomics, proteomics and metabolomics analyses of these lines might also shed light on potential biochemical pathways that are conferring tolerance to elevated [O_3_] in B73 × Mo17.

## CONFLICT OF INTEREST

The authors declare that they have no competing financial interests as defined by *Plant*, *Cell and Environment*, or other interests that might be perceived to influence the results and/or discussion reported in this article. Any opinions, findings and conclusions or recommendations expressed in this publication are those of the author(s) and do not necessarily reflect the views of the U.S. Department of Agriculture. Mention of trade names or commercial products in this publication is solely for the purpose of providing specific information and does not imply recommendation or endorsement by the U.S. Department of Agriculture. USDA is an equal opportunity provider and employer.

## AUTHOR CONTRIBUTIONS

Nicole E. Choquette and Elizabeth A. Ainsworth conceived of and designed the original research plans; Nicole E. Choquette sampled the plants, measured the physiological and biochemical responses; Nicole E. Choquette, William Bezodis, and Amanda P. Cavanagh performed Rubisco experiments. Nicole E. Choquette and Elizabeth A. Ainsworth performed quality control analyses and statistical analysis; Nicole E. Choquette, Elizabeth A. Ainsworth and Amanda P. Cavanagh wrote the article.

## Supporting information


**Data S1.** Supporting Information.

## References

[pce13876-bib-0001] Ainsworth, E. A. (2017). Understanding and improving global crop response to ozone pollution. The Plant Journal, 90, 886–897. 27739639 10.1111/tpj.13298

[pce13876-bib-0002] Ainsworth, E. A. , & Gillespie, K. M. (2007). Estimation of total phenolic content and other oxidation substrates in plant tissues using Folin‐Ciocalteu reagent. Nature Protocols, 2(4), 875–877. 17446889 10.1038/nprot.2007.102

[pce13876-bib-0003] Ainsworth, E. A. , Yendrek, C. R. , Sitch, S. , Collins, W. J. , & Emberson, L. D. (2012). The effects of tropospheric ozone on net primary productivity and implications for climate change. Annual Review of Plant Biology, 63, 637–663. 10.1146/annurev-arplant-042110-103829 22404461

[pce13876-bib-0004] Ashmore, M. R. (2005). Assessing the future global impacts of ozone on vegetation. Plant, Cell and Environment, 28, 949–964.

[pce13876-bib-0005] Avnery, S. , Mauzerall, D. L. , Liu, J. , & Horowitz, L. W. (2011). Global crop yield reductions due to surface ozone exposure: 1. Year 2000 crop production losses and economic damage. Atmospheric Environment, 45, 2284–2296.

[pce13876-bib-0006] Betzelberger, A. M. , Gillespie, K. M. , McGrath, J. M. , Koester, R. P. , Nelson, R. L. , & Ainsworth, E. A. (2010). Effects of chronic elevated ozone concentration on antioxidant capacity, photosynthesis and seed yield of 10 soybean cultivars. Plant, Cell & Environment, 33, 1569–1581. 10.1111/j.1365-3040.2010.02165.x 20444212

[pce13876-bib-0007] Brendley, B. W. , & Pell, E. J. (1998). Ozone‐induced changes in biosynthesis of Rubisco and associated compensation to stress in foliage of hybrid poplar. Tree Physiology, 18, 81–90. 12651392 10.1093/treephys/18.2.81

[pce13876-bib-0008] Butz, N. D. , & Sharkey, T. D. (1989). Activity ratios of ribulose‐1, 5‐bisphosphate carboxylase accurately reflect carbamylation ratios. Plant Physiology, 89, 735–739. 16666614 10.1104/pp.89.3.735 PMC1055915

[pce13876-bib-0009] Carmo‐Silva, A. E. , Keys, A. J. , Andralojc, P. J. , Powers, S. J. , Arrabaça, M. C. , & Parry, M. A. J. (2010). Rubisco activities, properties, and regulation in three different C_4_ grasses under drought. Journal of Experimental Botany, 61, 2355–2366. 20363871 10.1093/jxb/erq071 PMC2877893

[pce13876-bib-0011] Choquette, N. E. , Ogut, F. , Wertin, T. M. , Montes, C. M. , Sorgini, C. A. , Morse, A. M. , … Ainsworth, E. A. (2019). Uncovering hidden genetic variation in photosynthesis of field‐grown maize under ozone pollution. Global Change Biology, 25, 4327–4338. 31571358 10.1111/gcb.14794 PMC6899704

[pce13876-bib-0014] Dann, M. S. , & Pell, E. J. (1989). Decline of activity and quantity of ribulose bisphosphate carboxylase/oxygenase and net photosynthesis in ozone‐treated potato foliage. Plant Physiology, 91, 427–432. 16667037 10.1104/pp.91.1.427 PMC1062010

[pce13876-bib-0015] Eckardt, N. A. , & Pell, E. J. (1994). O3‐induced degradation of Rubisco protein and loss of Rubisco mRNA in relation to leaf age in *Solatium tuberosum* L. New Phytologist, 127, 741–748. 10.1111/j.1469-8137.1994.tb02978.x 33874385

[pce13876-bib-0016] Edwards, G. E. , Furbank, R. T. , Hatch, M. D. , & Osmond, C. B. (2001). What does it take to be C_4_? Lessons from the evolution of photosynthesis. Plant Physiology, 125, 46–49. 11154293 10.1104/pp.125.1.46 PMC1539322

[pce13876-bib-0017] Emberson, L. D. , Pleijel, H. , Ainsworth, E. A. , van den Berg, M. , Ren, W. , Osborne, S. , … Van Dingenen, R. (2018). Ozone effects on crops and consideration in crop models. European Journal of Agronomy, 100, 19–34.

[pce13876-bib-0018] Enyedi, A. J. , Eckardt, N. A. , & Pell, E. J. (1992). Activity of ribulose bisphophate carboxylase/oxygenase from potato cultivars with differential response to ozone stress. New Phytologist, 122, 493–500. 10.1111/j.1469-8137.1992.tb00078.x 33874212

[pce13876-bib-0019] Farquhar, G. D. , & Sharkey, T. D. (1982). Stomatal conductance and photosynthesis. Annual Review of Plant Physiology and Plant Molecular Biology, 33, 317–345.

[pce13876-bib-0020] Feller, U. , Anders, I. , & Mae, T. (2008). Rubiscolytics: Fate of Rubisco after its enzymatic function in a cell is terminated. Journal of Experimental Botany, 59(7), 1615–1624. 17975207 10.1093/jxb/erm242

[pce13876-bib-0021] Feng, Z. , Kobayashi, K. , & Ainsworth, E. A. (2008). Impact of elevated ozone concentration on growth, physiology, and yield of wheat (*Triticum aestivum* L.): A meta‐analysis. Global Change Biology, 14, 2696–2708.

[pce13876-bib-0022] Feng, Z. , Pang, J. , Kobayashi, K. , Zhu, J. , & Ort, D. R. (2011). Differential responses in two varieties of winter wheat to elevated ozone concentration under fully open‐air field conditions. Global Change Biology, 17, 580–591.

[pce13876-bib-0024] Fiscus, E. L. , Brooker, F. L. , & Burkey, K. O. (2005). Crop responses to ozone: Uptake, modes of action, carbon assimilation and partitioning. Plant, Cell and Environment, 28, 997–1011.

[pce13876-bib-0025] Food and Agriculture Organization . (2018). FAOSTAT, Retrieved from http://www.fao.org/faostat/en/#home

[pce13876-bib-0026] Foyer, C. H. , & Noctor, G. (2005). Redox homeostasis and antioxidant signaling: A metabolic interface between stress perception and physiological responses. Plant Cell, 17, 1866–1875. 15987996 10.1105/tpc.105.033589 PMC1167537

[pce13876-bib-0027] Furbank, R. T. , & Hatch, M. D. (1987). Mechanism of C_4_ photosynthesis. The size and composition of the inorganic carbon pool in bundle sheath cells. Plant Physiology, 85, 958–964. 16665838 10.1104/pp.85.4.958 PMC1054376

[pce13876-bib-0028] Galant, A. , Koester, R. P. , Ainsworth, E. A. , Hicks, L. M. , & Jez, J. M. (2012). From climate change to molecular response: Redox proteomics of ozone‐induced responses in soybean. New Phytologist, 194, 220–229. 10.1111/j.1469-8137.2011.04037.x 22272738

[pce13876-bib-0029] Galmés, J. , Aranjuelo, I. , Medrano, H. , & Flexas, J. (2013). Variation in Rubisco content and activity under variable climatic factors. Photosynthesis Research, 117(1–3), 73–90. 23748840 10.1007/s11120-013-9861-y

[pce13876-bib-0030] Galmés, J. , Conesa, M. A. , Ochogavia, J. M. , Perdomo, J. A. , Francis, D. M. , Ribas‐Carbo, M. , … Cifre, J. (2011). Physiological and morphological adaptations in relation to water use efficiency in Mediterranean accessions of *Solanum lycopersicum* . Plant Cell and Environment, 34, 245–260. 10.1111/j.1365-3040.2010.02239.x 20955222

[pce13876-bib-0031] Garcia‐Ferris, C. , & Moreno, J. (1993). Redox regulation of enzymatic activity and proteolytic susceptibility of ribulose‐ 1,5‐bisphosphate carboxylase/oxygenase from *Euglena gracilis* . Photosynthesis Research, 35, 55–66. 24318620 10.1007/BF02185411

[pce13876-bib-0032] Garcia‐Ferris, C. , & Moreno, J. (1994). Oxidative modification and breakdown of ribulose‐1,5‐bisphosphate carboxylase/oxygenase induced in *Euglena gracilis* by nitrogen starvation. Planta, 193().208–215.

[pce13876-bib-0033] Ghannoum, O. , Evans, J. R. , Chow, W. S. , Andrews, T. J. , Conroy, J. P. , & von Caemmerer, S. (2005). Faster Rubisco is the key to superior nitrogen‐use efficiency in NADP‐malic enzyme relative to NAD‐malic enzyme C_4_ grasses. Plant Physiology, 137(2), 638–350. 15665246 10.1104/pp.104.054759 PMC1065364

[pce13876-bib-0034] Gillespie, K. M. , & Ainsworth, E. A. (2007). Measurement of reduced, oxidized and total ascorbate content in plants. Nature Protocols, 2(4), 871–874. 17446888 10.1038/nprot.2007.101

[pce13876-bib-0035] Gillespie, K. M. , Rogers, A. , & Ainsworth, E. A. (2011). Growth at elevated ozone or elevated carbon dioxide concentration alters antioxidant capacity and response to acute oxidative stress in soybean (*Glycine max*). Journal of Experimental Botany, 62(8), 2667–2678. 21282325 10.1093/jxb/erq435

[pce13876-bib-0036] Goumenaki, E. , Taybi, T. , Borland, A. , & Barnes, J. (2010). Mechanisms underlying the impacts of ozone on photosynthetic performance. Environmental and Experimental Botany, 69, 259–266.

[pce13876-bib-0037] Grace, S. C. , & Logan, B. A. (2000). Energy dissipation and radical scavenging by the plant phenylpropanoid pathway. Philosophical Transactions of the Royal Society of London‐Biological Sciences, 355, 1499–1510. 10.1098/rstb.2000.0710 PMC1692864 11128003

[pce13876-bib-0038] Grantz, D. A. , & Vu, H. B. (2009). O_3_ sensitivity in a potential C_4_ bioenergy crop: Sugarcane in California. Crop Science, 49, 643–650.

[pce13876-bib-0039] Grantz, D. A. , Vu, H. B. , Tew, T. L. , & Veremis, J. C. (2012). Sensitivity of gas exchange parameters to ozone in diverse C_4_ sugarcane hybrids. Crop Science, 52, 1270–1280.

[pce13876-bib-0040] Heagle, A. S. , Kress, L. W. , Temple, P. J. , Kohut, R. J. , Miller, J. E. , & Heggestad, H. E. (1988). Ozone dose yield response relationships In Heck W. W., Taylor O. C., & Tingey D. T. (Eds.), Assessment of crop loss from air pollutants (pp. 141–179). London, England; New York, NY: Elsevier Applied Science.

[pce13876-bib-0041] Heath, R. L. (2008). Modification of the biochemical pathways of plants induced by ozone: What are the varied routes to change? Environmental Pollution, 155(3), 453–463. 18456378 10.1016/j.envpol.2008.03.010

[pce13876-bib-0042] Huffaker, R. C. (1982). Biochemistry and physiology of leaf proteins. *Encyclopedia of Plant Physiology* In Nucleic acids and proteins in plants, 14, 370–400). Berlin, Heidelberg: ).Springer.

[pce13876-bib-0044] Junqua, M. , Biolley, J.‐P. , Pie, S. , Kanoun, M. , Duran, R. , & Goulas, P. (2000). *In vivo* occurrence of carbonyl residues in *Phaseolus vulgaris* proteins as a direct consequence of a chronic ozone stress. Plant Physiology and Biochemistry, 38, 853–861.

[pce13876-bib-0045] Kane, H. J. , Wilkin, J.‐M. , Portis, A. R. , & Andrews, T. J. (1998). Potent inhibition of ribulose‐bisphosphate carboxylase by an oxidized impurity in ribulose‐1,5‐bisphosphate. Plant Physiology, 117, 1059–1069. 9662549 10.1104/pp.117.3.1059 PMC34922

[pce13876-bib-0046] Kangasjärvi, J. , Jaspers, P. , & Kollist, H. (2005). Signalling and cell death in ozone‐exposed plants. Plant Cell and Environment, 28, 1021–1037.

[pce13876-bib-0047] Kangasjärvi, J. , Talvinen, J. , Utriainen, M. , & Karjalainen, R. (1994). Plant defence systems induced by ozone. Plant Cell and Environment, 17, 783–794.

[pce13876-bib-0048] Kingston‐Smith, A. H. , & Foyer, C. H. (2000). Bundle sheath proteins are more sensitive to oxidative damage than those of the mesophyll in maize leaves exposed to paraquat or low temperatures. Journal of Experimental Botany, 51, 123–130. 10938803

[pce13876-bib-0049] Kingston‐Smith, A. H. , Harbinson, J. , & Foyer, C. H. (1999). Acclimation of photosynthesis, H_2_O_2_ content and antioxidants in maize (*Zea mays*) grown at sub‐optimal temperatures. Plant Cell and Environment, 22, 1071–1083.

[pce13876-bib-0050] Koricheva, J. , Larsson, S. , Haukioja, E. , & Keinanen, M. (1998). Regulation of woody plant secondary metabolism by resource availability: Hypothesis testing by means of meta‐analysis. Oikos, 83(2), 212–226.

[pce13876-bib-0051] Kubien, D. S. , Brown, C. M. , & Kane, H. J. (2011). Quantifying the amount and activity of Rubisco in leaves In Photosynthesis research protocols (pp. 349–362). Totowa, NJ.: Chap. 27.Humana Press. 10.1007/978-1-60761-925-3_27 20960142

[pce13876-bib-0053] Leisner, C. P. , & Ainsworth, E. A. (2012). Quantifying the effects of ozone on plant reproductive growth and development. Global Change Biology, 18, 606–616.

[pce13876-bib-0054] Leitao, L. , Bethenod, O. , & Biolley, J. (2007). The impact of ozone on juvenile maize (*Zea mays* L.) plant photosynthesis: Effects on vegetative biomass, pigmentation, and carboxylases (PEPc and Rubisco). Plant Biology, 9, 478–488. 17401809 10.1055/s-2007-964942

[pce13876-bib-0055] Leitao, L. , Maoret, J. J. , & Biolley, J.‐P. (2007). Changes in PEP carboxylase, Rubisco and Rubisco activase mRNA levels from maize (*Zea mays*) exposed to a chronic ozone stress. Biological Research, 40, 137–153. 18064351 10.4067/s0716-97602007000200005

[pce13876-bib-0056] Li, G. M. , Shi, Y. , & Chen, X. (2008). Effects of elevated carbon dioxide and ozone on the growth and secondary metabolism of spring wheat. Journal of Applied Ecology, 19, 1283–1288. 18808021

[pce13876-bib-0057] Li, P. , Calatayud, V. , Gao, F. , Uddling, J. , & Feng, Z. (2016). Differences in ozone sensitivity among woody species are related to leaf morphology and antioxidant levels. Tree Physiology, 36, 1105–1116. 27217527 10.1093/treephys/tpw042

[pce13876-bib-0058] Li, S. , Courbet, G. , Ourry, A. , & Ainsworth, E. A. (2019). Elevated ozone concentration reduces photosynthetic carbon gain but does not alter leaf structural traits, nutrient composition or biomass in switchgrass. Plants, 8, 85 10.3390/plants8040085 PMC6524373 30987071

[pce13876-bib-0059] Lim, P. O. , Kim, H. J. , & Nam, H. G. (2007). Leaf senescence. Annual Review of Plant Biology, 58, 115–136. 10.1146/annurev.arplant.57.032905.105316 17177638

[pce13876-bib-0060] Liu, T. , Ren, T. , White, P. J. , Cong, R. , & Lu, J. (2018). Storage nitrogen co‐ordinates leaf expansion and photosynthetic capacity in winter oilseed rape. Journal of Experimental Biology, 69(12), 2995–3007. 10.1093/jxb/ery134 PMC5972566 29669007

[pce13876-bib-0061] Luwe, M. , Takahama, U. , & Heber, U. (1993). Role of ascorbate in detoxifying ozone in the apoplast of spinach (*Spinacia oleracea* L.) leaves. Plant Physiology, 101, 969–976. 12231749 10.1104/pp.101.3.969 PMC158714

[pce13876-bib-0062] McGrath, J. M. , Betzelberger, A. M. , Wang, S. , Shook, E. , Zhu, X.‐G. , Long, S. P. , & Ainsworth, E. A. (2015). An analysis of ozone damage to historical maize and soybean yields in the United States. Proceedings of the National Academy of Sciences, 112(46), 14390–14395. 10.1073/pnas.1509777112 PMC4655515 26578785

[pce13876-bib-0063] Millard, P. , & Grelet, G. A. (2010). Nitrogen storage and remobilization by trees: Ecophysiological relevance in a changing world. Tree Physiology, 30, 1083–1095. 20551251 10.1093/treephys/tpq042

[pce13876-bib-0064] Miller, J. D. , Arteca, R. N. , & Pell, E. J. (1999). Senescence‐associated gene expression during ozone‐induced leaf senescence in Arabidopsis. Plant Physiology, 120, 1015–1023. 10444084 10.1104/pp.120.4.1015 PMC59334

[pce13876-bib-0065] Miller, J. E. (1988). Effects on photosynthesis, carbon allocation and plant growth associated with air pollutant stress In Heck W. W., Taylor O. C., & Tingey D. T. (Eds.), Assessment of crop loss from air pollutants (pp. 287–314). London, England; New York, NY: Elsevier Applied Science.

[pce13876-bib-0067] Mills, G. , Pleijel, H. , Malley, C. S. , Sinha, B. , Cooper, O. R. , Schultz, M. G. , … Xu, X. (2018). Tropospheric ozone assessment report: Present‐day tropospheric ozone distribution and trends relevant to vegetation. Elementa Science of the Anthropocene, 6, 47.

[pce13876-bib-0068] Mills, G. , Sharps, K. , Simpson, D. , Pleijel, H. , Brogery, M. , Uddling, J. , … Van Dingenen, R. (2018b). Ozone pollution will compromise efforts to increase global wheat production. Global Change Biology, 24, 3560–3574. 29604158 10.1111/gcb.14157

[pce13876-bib-0069] Mills, G. , Sharps, K. , Simpson, D. , Pleijel, H. , Frei, M. , Burkey, K. , … Agrawal, M. (2018c). Closing the global ozone yield gap: Quantification and co‐benefits for multistress tolerance. Global Change Biology, 24, 4869–4893. 30084165 10.1111/gcb.14381

[pce13876-bib-0070] Morgan, P. B. , Ainsworth, E. A. , & Long, S. P. (2003). How does elevated ozone impact soybean? A meta‐analysis of photosynthesis, growth and yield. Plant Cell and Environment, 26, 1317–1328.

[pce13876-bib-0102] Morgan, P.B. , Bernacchi, C.J. , Ort, D.R., & , & Long, S.P (2004). An in vivo analysis of the effect of season‐long open‐air elevation of ozone to anticipated 2050 levels on photosynthesis in soybean. Plant Physiology, 235, 2348–2357. 10.1104/pp.104.043968 PMC520802 15299126

[pce13876-bib-0071] Pang, J. , Kobayashi, K. , & Zhu, J. (2009). Photosynthetic characteristics of flag leaves in Chinese rice (*Oryza sativa* L.) varieties subjected to free‐air release of ozone. Agriculture, Ecosystems, and Environment, 132, 203–211.

[pce13876-bib-0072] Pell, E. J. , & Pearson, N. S. (1983). Ozone‐induced reduction in quantity of ribulose‐1,5‐ bisphosphate carboxylase in alfalfa foliage. Plant Physiology, 73, 185–187. 16663173 10.1104/pp.73.1.185 PMC1066432

[pce13876-bib-0073] Pelloux, J. , Jolivet, Y. , Fontaine, V. , Banvoy, J. , & Dizengremel, P. (2001). Changes in Rubisco and Rubisco activase gene expression and polypeptide content in *Pinus halepensis* M. subjected to ozone and drought. Plant Cell and Environment, 24, 123–131.

[pce13876-bib-0074] Peltonen, P. A. , Vapaavuori, E. , & Julkunen‐Tiitto, R. (2005). Accumulation of phenolic compounds in birch leaves is changed by elevated carbon dioxide and ozone. Global Change Biology, 11, 1305–1324.

[pce13876-bib-0075] Perdomo, J. A. , Capo‐Bauca, S. , Carmo‐Silva, E. , & Galmes, J. (2017). Rubisco and Rubisco activase play an important role in the biochemical limitations of photosynthesis in rice, wheat, and maize under high temperature and water deficit. Frontiers in Plant Science, 8, 1–15. 28450871 10.3389/fpls.2017.00490 PMC5390490

[pce13876-bib-0077] Pierce, J. , Tolbert, N. E. , & Barker, R. (1980). Interaction of ribulose bisphosphate carboxylase/oxygenase with transition‐state analogs. Biochemistry, 19, 934–942. 7356969 10.1021/bi00546a018

[pce13876-bib-0078] Portis, A. R., Jr. (2003). Rubisco activase – Rubisco's catalytic chaperone. Photosynthesis Research, 75, 11–27. 16245090 10.1023/A:1022458108678

[pce13876-bib-0079] Reid, C. D. , Fiscus, E. L. , & Burkey, K. O. (1998). Combined effects of chronic ozone and elevated CO_2_ on Rubisco activity and leaf components in soybean (*Glycine max*). Journal of Experimental Botany, 49, 1999–2011.

[pce13876-bib-0080] Robinson, S. P. , & Portis, A. R., Jr. (1988). Release of the nocturnal inhibitor, carboxyarabinitol‐1‐phosphate from ribulose bisphosphate carboxylase/oxygenase by Rubisco activase. FEBS Letters, 233, 413–416.

[pce13876-bib-0081] Ruuska, S. , Andrews, T. J. , Badger, M. R. , Hudson, G. S. , Laisk, A. , Price, G. D. , & von Caemmerer, S. (1998). The interplay between limiting processes in C_3_ photosynthesis studied by rapid‐response gas exchange using transgenic tobacco impaired in photosynthesis. Australian Journal of Plant Physiology, 25, 859–870.

[pce13876-bib-0082] Sage, R. F. , & Pearcy, R. W. (1987). The nitrogen use efficiency of C_3_ and C_4_ plants. Plant Physiology, 84, 959–963. 16665551 10.1104/pp.84.3.959 PMC1056702

[pce13876-bib-0084] Salesse‐Smith, C. E. , Sharwood, R. E. , Busch, R. A. , Kromdijk, J. , Bardal, V. , & Stern, D. B. (2018). Overexpression of Rubisco subunits with RAF1 increases Rubisco content in maize. Nature Plants, 4, 802–810. 30287949 10.1038/s41477-018-0252-4

[pce13876-bib-0087] Scebba, F. , Pucciarelli, I. , Soldatini, G. F. , & Ranieri, A. (2003). O_3_‐induced changes in the antioxidant systems and their relationship to different degrees of susceptibility of two clover. Plant Science, 165, 583–593.

[pce13876-bib-0088] Sharwood, R. E. , Sonawane, B. V. , Ghannoum, O. , & Whitney, S. M. (2016). Improved analysis of C_4_ and C_3_ photosynthesis via refined *in vitro* assays of their carbon fixation biochemistry. Journal of Experimental Botany, 67(10), 3137–3148. 27122573 10.1093/jxb/erw154 PMC4867899

[pce13876-bib-0089] Singh, A. A. , Agrawal, S. B. , Shahi, J. P. , & Agrawal, M. (2014). Investigating the response of tropical maize (*Zea mays* L.) cultivars against elevated levels of O_3_ at two developmental stages. Ecotoxicology, 23, 1447–1463. 25023387 10.1007/s10646-014-1287-6

[pce13876-bib-0090] Tai, A. P. , Martin, M. V. , & Heald, C. L. (2014). Threat to future global food security from climate change and ozone air pollution. Nature Climate Change, 4, 817–821.

[pce13876-bib-0091] Van Dingenen, R. , Dentener, F. J. , Raes, F. , Krol, M. C. , Emberson, L. , & Cofala, J. (2009). The global impact of ozone on agricultural crop yields under current and future air quality legislation. Atmospheric Environment, 43(3), 604–618.

[pce13876-bib-0092] von Caemmerer, S. (2000). Biochemical models of leaf photosynthesis, Techniques in Plant Sciences, (Vol. 2, Collingwood VIC Australia: ). CSIRO Publishing.

[pce13876-bib-0093] von Caemmerer, S. , & Furbank, R. T. (2003). The C_4_ pathway, an efficient CO_2_ pump. Photosynthesis Research, 77, 191–207. 16228376 10.1023/A:1025830019591

[pce13876-bib-0094] von Caemmerer, S. , Hendrickson, L. , Quinn, V. , Vella, N. , Millgate, A. G. , & Furbank, R. T. (2005). Reductions of Rubisco activase by antisense RNA in the C_4_ plant *Flaveria bidentis* reduces Rubisco carbamylation and leaf photosynthesis. Plant Physiology, 137, 747–755. 15665240 10.1104/pp.104.056077 PMC1065374

[pce13876-bib-0095] von Caemmerer, S. , Millgate, A. , Farquhar, G. D. , & Furbank, R. T. (1997). Reduction of ribulose‐1,5‐bisphosphate carboxylase/oxygenase by antisense RNA in the C_4_ plant *Flaveria bidentis* leads to reduced assimilation rates and increased carbon isotope discrimination. Plant Physiology, 113, 469–477. 12223620 10.1104/pp.113.2.469 PMC158162

[pce13876-bib-0096] Wellburn, F. A. M. , & Wellburn, A. R. (1996). Variable patterns of antioxidant protection but similar ethene emission differences in several ozone‐sensitive and ozone‐tolerant plant selections. Plant, Cell and Environment, 19, 754–760.

[pce13876-bib-0098] Yendrek, C. R. , Erice, G. , Montes, C. M. , Tomaz, T. , Sorgini, C. A. , Brown, P. J. , … Ainsworth, E. A. (2017). Elevated ozone reduces photosynthetic carbon gain by accelerating leaf senescence of inbred and hybrid maize in a genotype‐specific manner. Plant, Cell Environment, 40, 3088–3100. 10.1111/pce.13075 29044553

[pce13876-bib-0099] Yendrek, C. R. , Koester, R. P. , & Ainsworth, E. A. (2015). A comparative analysis of transcriptomic, biochemical, and physiological responses to elevated ozone identifies species‐specific mechanisms of resilience in legume crops. Journal of Experimental Botany, 66, 7101–7112. 26324463 10.1093/jxb/erv404 PMC4765784

[pce13876-bib-0100] Yendrek, C. R. , Leisner, C. P. , & Ainsworth, E. A. (2013). Chronic ozone exacerbates the reduction in photosynthesis and acceleration of senescence caused by limited N availability in *Nicotiana sylvestris* . Global Change Biology, 19, 3155–3166. 23625780 10.1111/gcb.12237

[pce13876-bib-0101] Yendrek, C. R. , Tomaz, T. , Montes, C. M. , Cao, Y. , Morse, A. M. , Brown, P. J. , … Ainsworth, E. A. (2017). High‐throughput phenotyping of maize leaf physiological and biochemical traits using hyperspectral reflectance. Plant Physiology, 173, 614–626. 28049858 10.1104/pp.16.01447 PMC5210743

