# Supplementary material for: Ozone tolerant maize hybrids maintain Rubisco content and activity during long‐term exposure in the field
Source: Plant Cell Environ. 2020 Oct 22;43(12):3033–47. doi: 10.1111/pce.13876 (PMC7756399; doi:10.1111/pce.13876)
Supplement: Supplementary file 1 — Data S1. Supporting Information. [file PCE-43-3033-s001.pdf]

Supplemental Table 1. Analysis of variance (F, p) of the maximum apparent rate of phosphoenolpyruvate carboxylase activity (V_{pmax}), CO_2-saturated photosynthetic rate (V_{max}), initial and total Rubisco activation and activation state measured for six different hybrids in ambient and elevated O_3 in June and July (n=4).

		June		July	
		F	p	F	p
V_{pmax}	Genotype	1.95	0.1098	0.9	0.4893
	O_3	29.35	<.0001	38.84	<.0001
	G x O_3	1.92	0.1145	3.07	0.0207
V_{max}	Genotype	0.83	0.5341	0.28	0.9214
	O_3	22.85	<.0001	20.69	<.0001
	G x O_3	1.26	0.3036	0.77	0.5786
Rubisco content	Genotype	3.37	0.0191	0.78	0.5714
	O_3	44.59	<0.0001	64.74	<0.0001
	G x O_3	0.83	0.5435	2.40	0.0670
Total soluble protein	Genotype	1.03	0.4235	0.96	0.4626
	O_3	1.01	0.3247	5.26	0.0322
	G x O_3	0.52	0.7587	1.13	0.3754
Carbamylation status	Genotype	3.44	0.0176	0.87	0.5138
	O_3	1.66	0.2096	0.21	0.6514
	G x O_3	1.98	0.1178	2.23	0.0842
In vitro Rubisco activity	Genotype	2.13	0.0864	3.67	0.0097
	O_3	0.31	0.5835	36.55	<0.0001
	G x O_3	2.02	0.1016	3.37	0.0148

Supplemental Table 2. Analysis of variance (F, p) of net photosynthesis (*A*), stomatal conductance (g_s), intercellular CO_2 (C_i), and stomatal limitation measured in six different hybrids in ambient and elevated O_3 in June and July (n=4).

		June		July	
		F	p	F	p
A	Genotype	2.1	0.0908	0.57	0.7233
	O_3	21.24	<.0001	32.22	<.0001
	G x O_3	1.16	0.3508	1.31	0.2842
g_s	Genotype	3.4	0.0138	0.6	0.7038
	O_3	11.5	0.0018	0.19	0.6629
	G x O_3	0.52	0.7624	1.05	0.4063
Ci	Genotype	3.83	0.0076	2.96	0.0256
	O_3	20.64	<.0001	69.39	<.0001
	G x O_3	3.53	0.0115	2.31	0.0662
Stomatal Limitation	Genotype	4.34	0.0038	1.46	0.228
	O_3	4.18	0.0491	3.93	0.0557
	G x O_3	0.47	0.7938	1.83	0.1335

Supplemental Table 3. Analysis of variance (F, p) of percent nitrogen (% N) and specific leaf area (SLA) measured in six different hybrids in ambient and elevated O_3 in June and July (n=4).

		June		July	
		F	p	F	p
% N	Genotype	1.63	0.1784	2.05	0.0974
	O_3	15.30	0.0004	56.19	<.0001
	G x O_3	0.15	0.9785	1.63	0.1797
SLA	Genotype	7.65	<.0001	0.59	0.7048
	O_3	1.14	0.2929	1.75	0.1947
	G x O_3	0.84	0.5307	0.27	0.9269

Supplemental Table 4. Analysis of variance (F, p) of chlorophyll a, b, carotenoids, and ratio of chlorophyll a to b (chl a / chl b) measured in six different hybrids in ambient and elevated O_3 in June and July (n=4).

		June		July	
		F	p	F	p
Chlorophyll a	Genotype	12.29	<.0001	7.23	0.0001
	O_3	27.82	<.0001	44.38	<.0001
	G x O_3	1.39	0.2547	1.42	0.2434
Chlorophyll b	Genotype	12.09	<.0001	4.66	0.0025
	O_3	27.47	<.0001	76.7	<.0001
	G x O_3	1.83	0.1333	1.31	0.2822
Carotenoids	Genotype	9.43	<.0001	—	—
	O_3	1.38	0.2492	—	—
	G x O_3	0.69	0.6352	—	—
Chl a / Chl b	Genotype	1.83	0.135	2.95	0.0263
	O_3	0.28	0.5995	52.68	<.0001
	G x O_3	1.01	0.4276	0.56	0.7321

Supplemental Table 5. Analysis of variance (F, p) of phenolic content, total ascorbate, %reduced ascorbate, total glutathione, and %reduced glutathione measured in six different hybrids in ambient and elevated O_3 in June and July (n=4).

		June		July	
		F	p	F	p
Phenolic Content	Genotype	1.32	0.2809	4.15	0.0049
	O3	7.06	0.0121	3.12	0.0867
	G x O3	1.02	0.4215	0.62	0.6843
Total Ascorbate	Genotype	1.69	0.1657	2.15	0.0837
	O_3	1.23	0.2757	0.49	0.4897
	G x O_3	0.48	0.7872	0.7	0.6298
%reduced Ascorbate	Genotype	3.46	0.0126	2.13	0.0862
	O_3	2.55	0.1196	12.24	0.0014
	G x O_3	1.11	0.375	0.79	0.5666
Total Glutathione	Genotype	8.94	<.0001	2.68	0.0385
	O_3	2.03	0.1632	19.75	<.0001
	G x O_3	0.72	0.612	2.06	0.0954
%reduced Glutathione	Genotype	0.54	0.7446	0.67	0.6525
	O_3	0.28	0.6011	1.52	0.227
	G x O_3	1.53	0.2083	1.48	0.2218

Supplemental Table 6. Analysis of variance (F, p) of glucose, fructose, and sucrose measured in six different hybrids in ambient and elevated O_3 in June and July (n=4).

		June		July	
		F	p	F	p
Glucose	Genotype	1.11	0.3744	2.8	0.0324
	O_3	2.46	0.1262	4.98	0.0326
	G x O_3	0.40	0.8459	0.68	0.6413
Fructose	Genotype	2.30	0.0670	3.28	0.0164
	O_3	11.29	0.0019	0.91	0.3466
	G x O_3	0.16	0.9743	1.92	0.1177
Sucrose	Genotype	3.62	0.0099	2.16	0.0830
	O_3	2.25	0.1432	3.40	0.0741
	G x O_3	0.82	0.5457	0.28	0.9183

Supplemental Table 7. Analysis of variance (F, p) of yield and individual kernel mass measured in six different hybrids in ambient and elevated O_3 (n=4).

	Yield		Kernel mass	
	F	p	F	p
Genotype	2.94	0.0266	1.46	0.2287
O_3	11.04	0.0022	7.78	0.0087
G x O_3	1.20	0.3322	0.25	0.9348

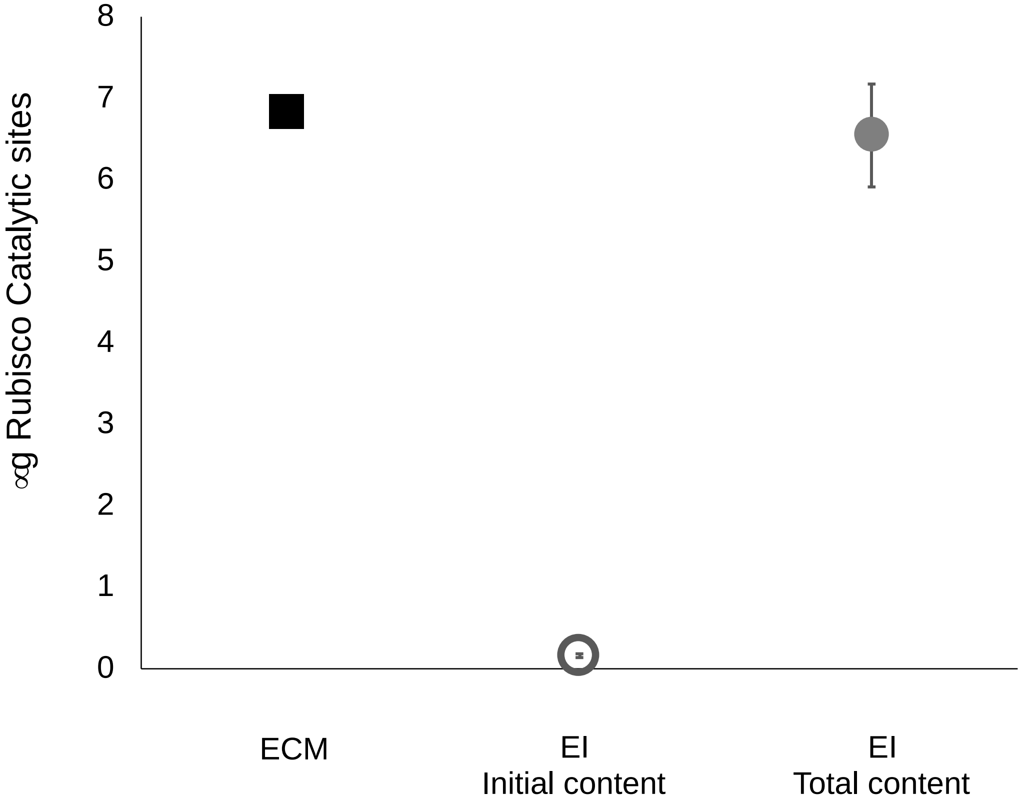

Supplemental Figure 1. Quantification of Rubisco catalytic sites of activated Rubisco (ECM; incubated with $MgCl_2$ and $NaHCO_3$), and inactivated Rubisco (EI, incubated in a buffer free of $MgCl_2$ and CO_2) before and after activating with $MgCl_2$ and $NaHCO_3$. Rubisco was isolated from *Arabidopsis thaliana* as described in Kim et al., 2019. Error bars represent standard deviation of the mean of n=3 replicates.

Citation: Kim, Sang Yeol, Christopher M. Harvey, Jonas Giese, Ines Lassowskat, Vijayata Singh, Amanda P. Cavanagh, Martin H. Spalding, Iris Finkemeier, Donald R. Ort, and Steven C. Huber. "In vivo evidence for a regulatory role of phosphorylation of Arabidopsis Rubisco activase at the Thr78 site." *Proceedings of the National Academy of Sciences* 116(37): 18723-18731.

DOI: https://doi.org/10.1073/pnas.1812916116

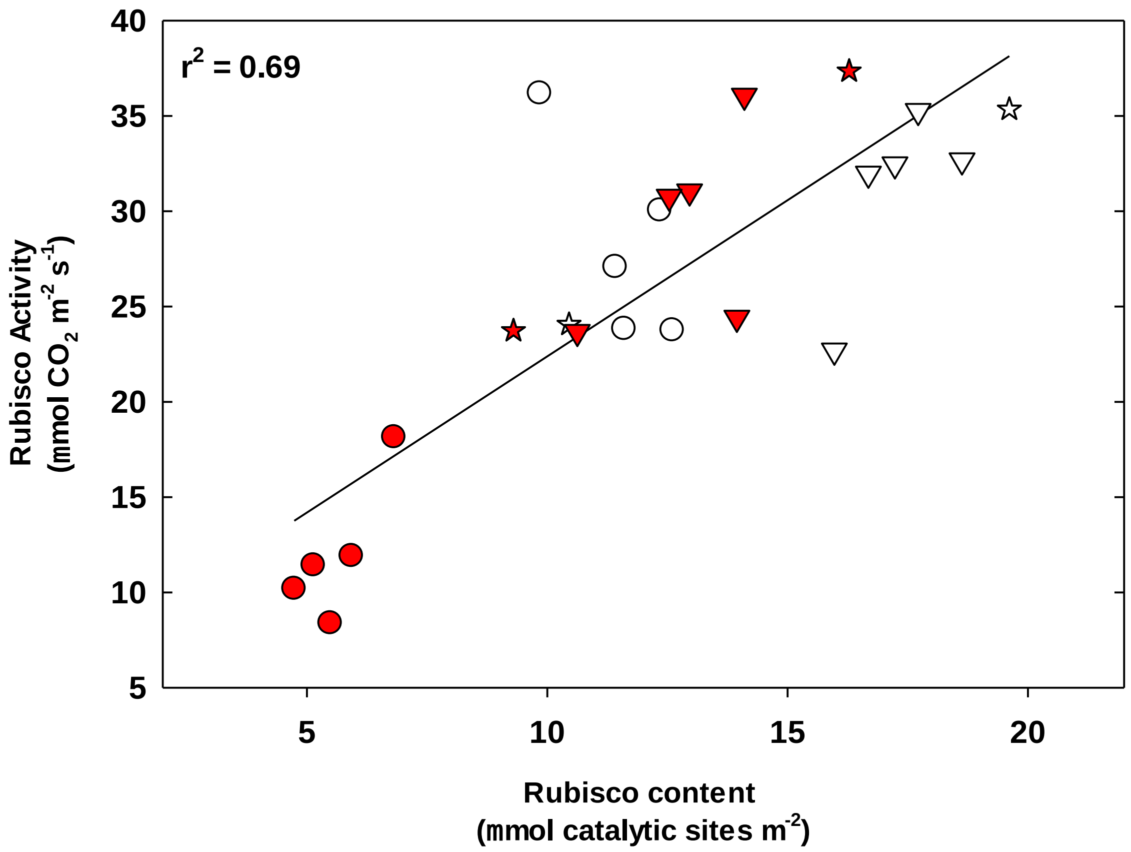

Supplemental Figure 2. Correlation between *in vitro* Rubisco activity and Rubisco content measured from size exclusion chromatography. Ambient ozone (white symbols); elevated ozone (red symbols); triangles – measurements in June; circles – measurements in July; stars indicate B73 x Mo17.
